# Supplementary material for: Structure of lymphostatin, a large multi-functional virulence factor of pathogenic Escherichia coli
Source: Nat Commun. 2025 Jun 25;16:5389. doi: 10.1038/s41467-025-60995-9 (PMC12198386; doi:10.1038/s41467-025-60995-9)
Supplement: Supplementary file 1 — Supplementary Information [file 41467_2025_60995_MOESM1_ESM.pdf]

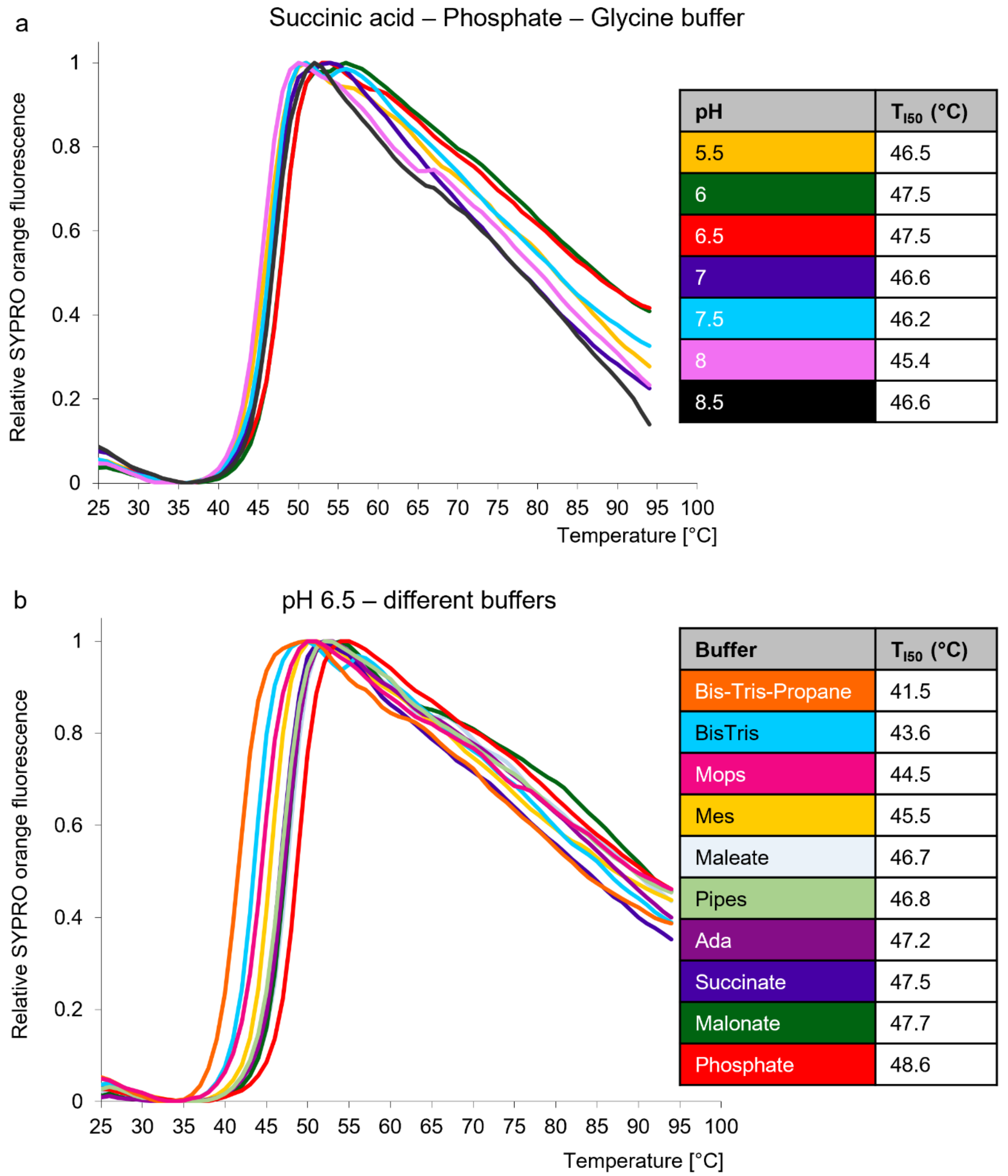

pH	T_{I50} (°C)
5.5	46.5
6	47.5
6.5	47.5
7	46.6
7.5	46.2
8	45.4
8.5	46.6

Buffer	T_{I50} (°C)
Bis-Tris-Propane	41.5
BisTris	43.6
Mops	44.5
Mes	45.5
Maleate	46.7
Pipes	46.8
Ada	47.2
Succinate	47.5
Malonate	47.7
Phosphate	48.6

Supplementary Figure 1 | Thermal Shift Assay of LifA at different pH and in different buffers. Temperature dependent unfolding of LifA was followed by monitoring the increase in fluorescence of added SYPRO orange. The fluorescence intensity of SYPRO orange increases when interacting with the hydrophobic core of an unfolded protein. **a**) The plot shows the temperature dependent increase in fluorescence in succinic-phosphate-glycine (SPG) in the range of pH 5.5-8.5. The table lists the pH values (background colour corresponds to the plot) together with the temperature at which the half maximal fluorescence intensity is reached (T_{i50}). The difference in T_{I50}) between the highest (pH 6.5 and 6.0) and the lowest (pH 8.0) T_{I50} is 2.1 K. **b**) Temperature dependent increase in fluorescence at pH 6.5 with different buffer systems. The table lists the buffer (background colour corresponds to the plot) together with the temperature at which the half maximal fluorescence intensity is reached (T_{I50}). The difference between the highest T_{I50} in phosphate buffer and the lowest T_{I50} in Bis-Tris-Propane buffer is 7.1 K. The T_{I50} was used as an estimate for the melting temperature T_m of LifA. The

Thermal shift assay was repeated 2 times independently (n=2). Source data are provided as a Source Data file.

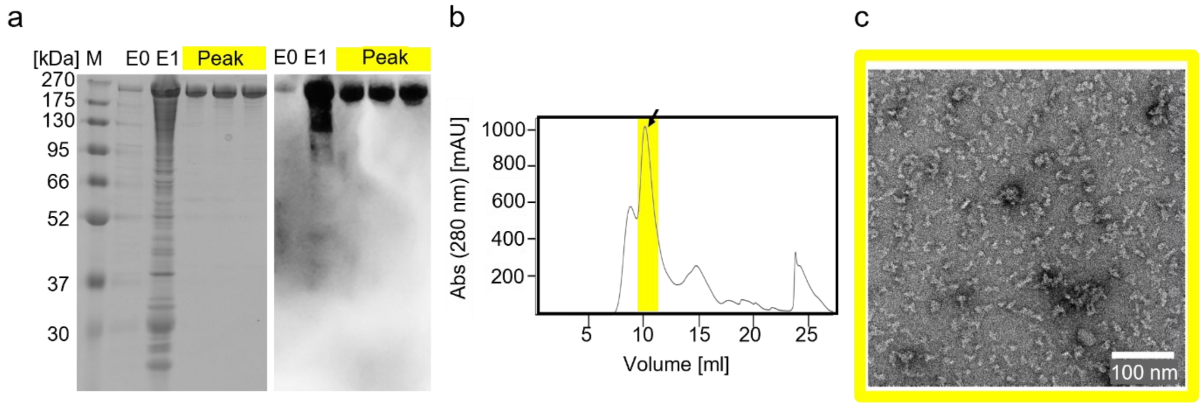

Supplementary Figure 2 | Protein purification of LifA. a) SDS-PAGE with Coomassie R-250 staining (left) and Western blot (right) of purified LifA. For the Western blot a penta-His antibody was used against the C-terminal His-Tag of LifA; M indicates the marker with the molecular weight indicated on the left. E0 and E1 are the elution fractions from the immobilized metal affinity chromatography (IMAC). The three peak fractions of the main peak of the SEC are labelled with “Peak”. **b)** Typical SEC-profile of LifA, where the elution fraction E1 of the IMAC was injected. LifA elutes after approx. 10 ml as indicated in yellow. **c)** Electron micrographs of negatively stained LifA (approx. 75 µg/ml) taken from the peak fractions indicated in **b**). The particles show different shapes and sizes up to approx. 20 nm as expected for L-shaped particles in different orientations. The purification was repeated at least three times with a similar outcome (n=3). Source data are provided as a Source Data file.

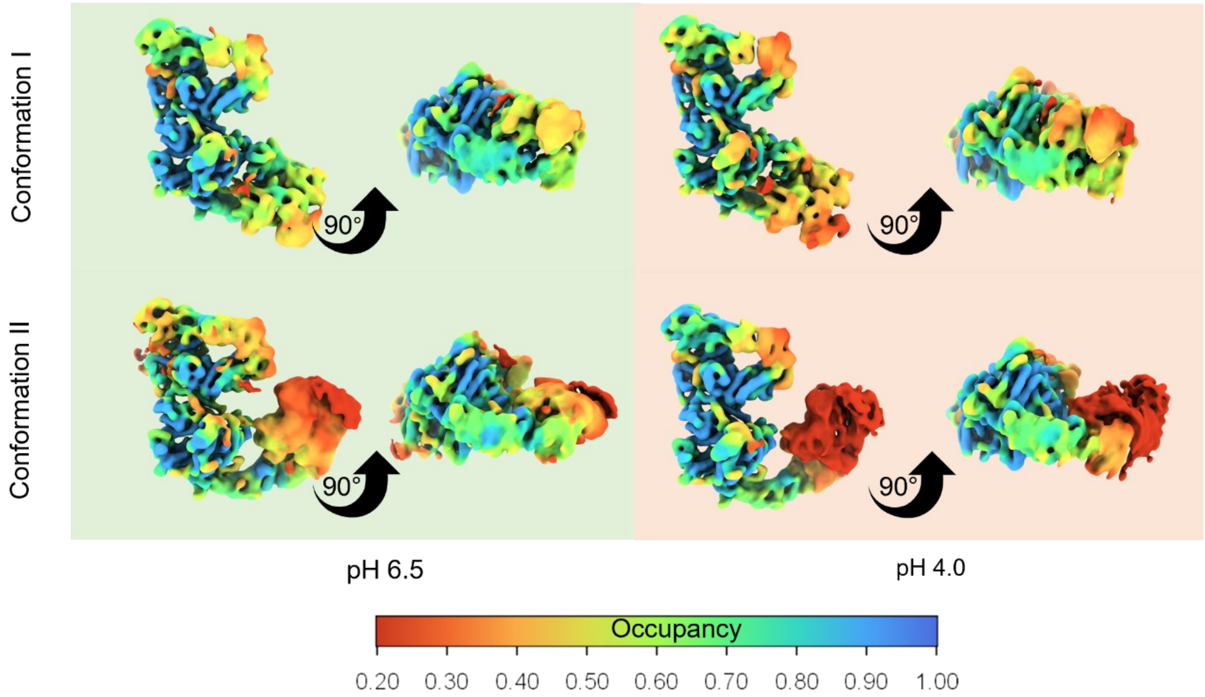

Supplementary Figure 3 | LifA adopts conformations I and II at pH 6.5 and at pH 4.0. Conformation I (upper panels) and II (lower panels) coexist at pH 6.5 and at pH 4.0: The surface representations were scaled and coloured with *OccuPy*[1]. All maps are aligned in respect to each other. The map of conformation I at pH 4.0 was low pass filtered to 8 Å (RELION_image_handler) and used as reference map for amplitude scaling of the other maps (RELION_image_handler). The grey values of the resulting maps were adjusted with *OccuPy* and the surface of the maps are coloured by the relative occupancy. The low occupancy in the C-terminal arm indicates high structural variability of the region. All maps are shown with the same threshold and were scaled with the same parameters in *OccuPy*. For each map two perpendicular views are shown.

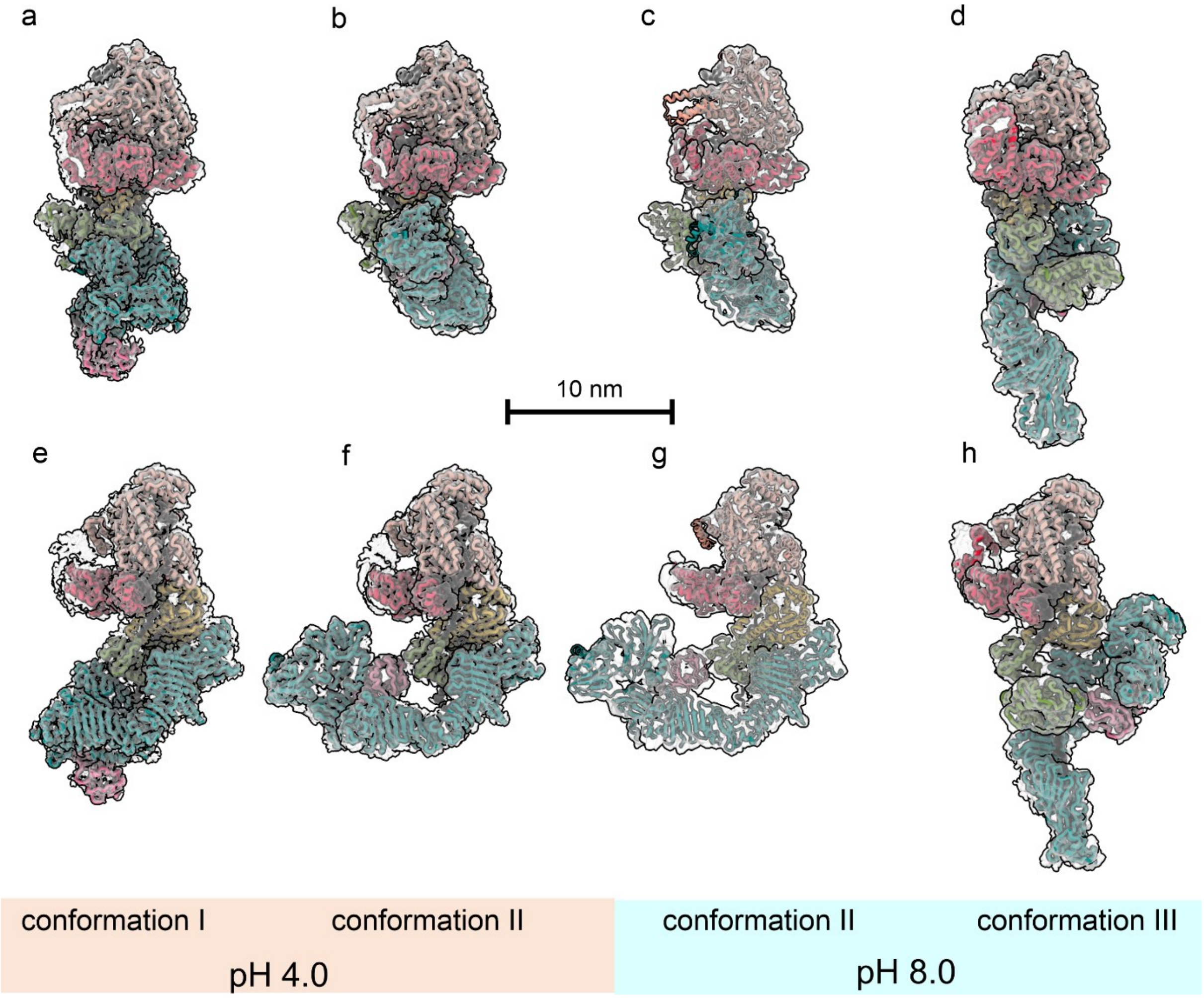

Supplementary Figure 4 | LifA adopts conformations I and II at pH 4.0 and conformations II and III at pH 8.0. The models are coloured according to the domains as shown in the main Figure 1 and are shown together with their respective combined focussed maps (transparent grey surface). Maps and models in **a**, **b**, **d** are rotated by 90° relative to the views shown in main Figure 1 and by 180° compared to those shown in **e**, **f** and **h**. The designated conformations are indicated below together with the pH value before vitrification. **c** and **g** show conformation II at pH 8.0 in phosphate buffer, which is not included in the main figure 1.

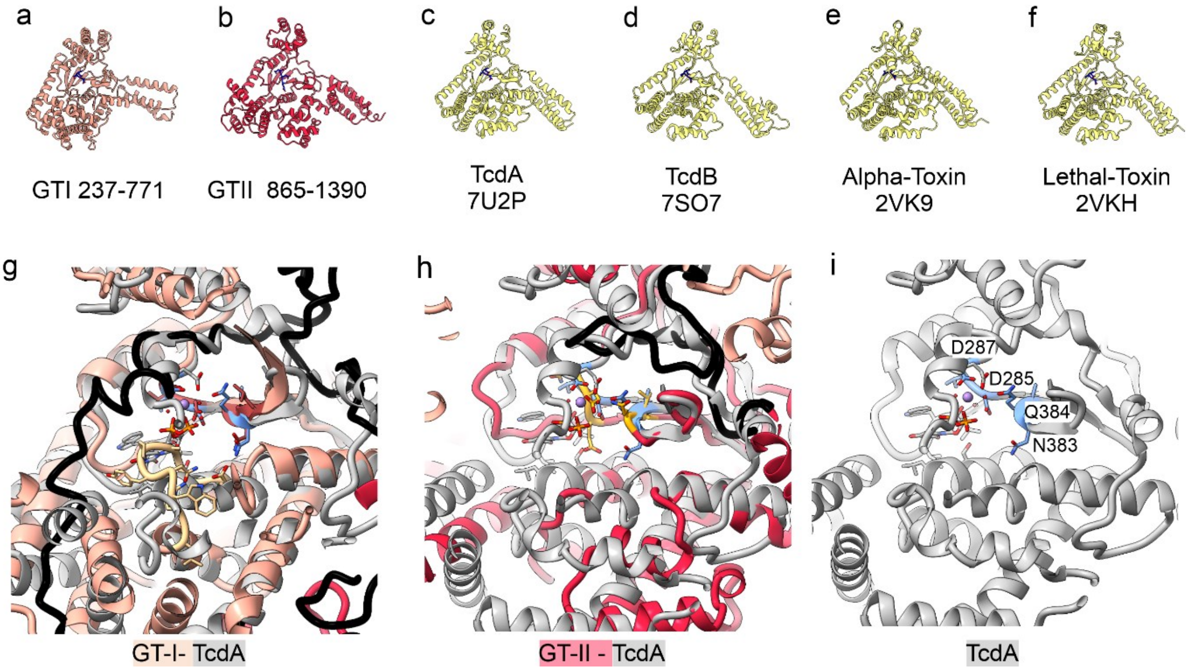

Supplementary Figure 5 | GT-I and GT-II have a similar fold as the glycolyl transferase domains of other large bacterial toxins. The Glycosyl transferase domains **a**) GT-I and **b**) GT-II are shown together with selected glycosyl transferase domains with known structure (**c-f**) that were identified as structural homologues of GT-II with FoldSeek [2]. All domains (**a** and **c-f**) were aligned to GT-II (b) with Matchmaker in Chimera X[3]. The 'EEN' motif of GT-II (**b**) and the 'DXD'-motif in all other GT-domains (**a** and **c-f**) are shown in blue. **c-f** show the match of the FoldSeek query and not the full structure of the toxin. The represented homologues are the GT-domains of the following LCTs: **c**) TcdA (pdb: 7U2P [4]), **d**) TcdB (pdb: 7SO7 [5]), **e**) Alpha toxin TcnA (pdb:2VK9 [6]) and **f**) Lethal-Toxin TcsL (pdb: 2VKH [6]).
g-i) View of the GT-I domain (**g**, pink) and the GT-II domain (**h**, red) as shown in Figure **2b** and **2d** with the UDP and Manganese bound GT-domain of TcdA superposed (white). **i**) The GT-domain of TcdA with bound Manganese and UDP (pdb: 4dMW [7]) as shown in **g**) and **h**).

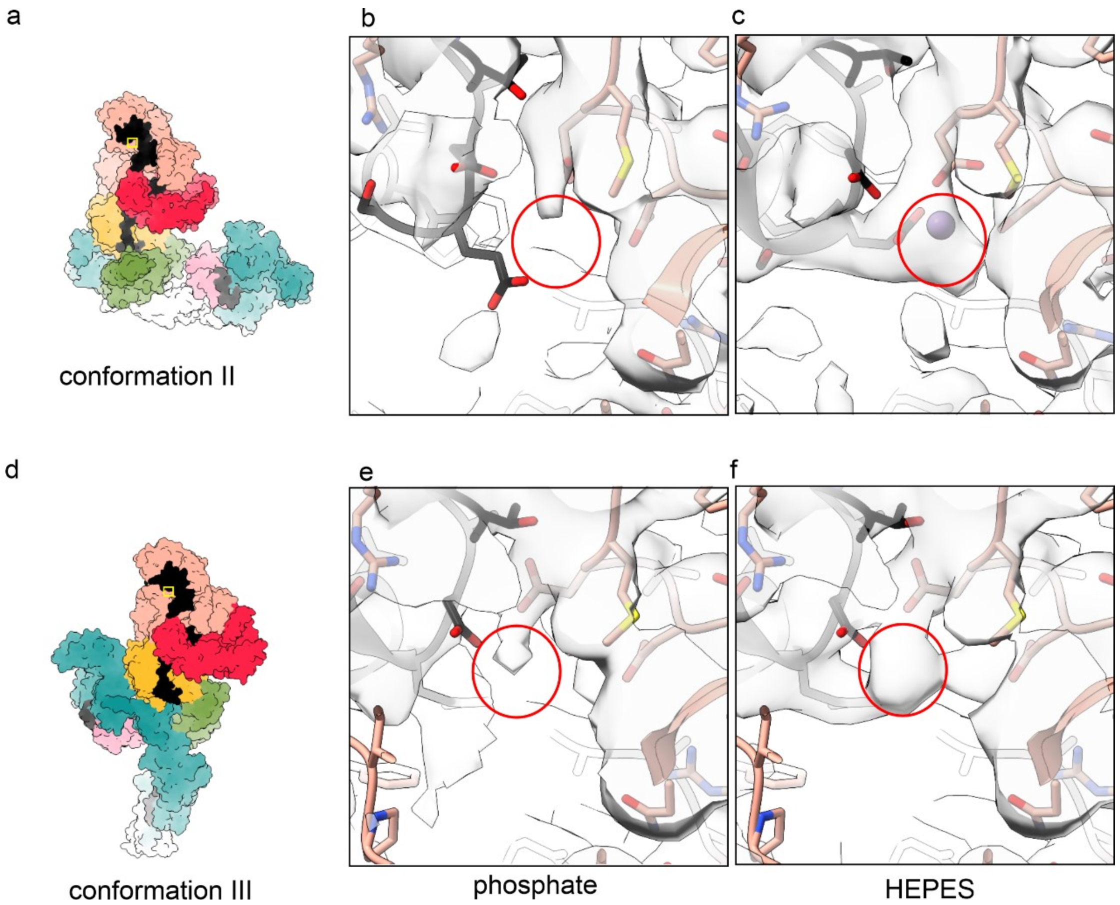

Supplementary Figure 6 | Density near the DXD-motif at the Mn^{2+}-binding site in GT-I at pH 8.0. **a-c**) Conformation II at pH 8.0. **a**) Model of conformation II at pH 8.0 in the same orientation as the close-ups in **b**) and **c**). The approximate position of the close-ups is indicated with a yellow square. **b**) and **c**) show close-ups of the maps and models at the potential Mn^{2+}-binding site. **b**) shows the map and model of conformation II of LifA in phosphate buffer and **c**) in HEPES buffer. In both maps/models the same position is indicated by a red circle and highlights the expected binding site of Mn^{2+}. There is a prominent density in HEPES buffer **c**), which was modelled as a Manganese ion (purple sphere). **b**) No density is present in phosphate buffer.

d-f) Conformation III at pH 8.0. **d**) The model of conformation III at pH 8.0 in the same orientation as the close-ups in **e**) and **f**). The model was built into the map derived from all particles at pH 8 (HEPES and phosphate buffer). The approximate position of the close-ups is indicated with a yellow square. **e**) The map was calculated from the subset of particles in phosphate buffer from the consensus refinement of all particles (HEPES and phosphate). **f**) The map was calculated from the subset of particles in HEPES buffer from the same consensus refinement. **e**) and **f**) show the same model. The red circle marks the same position in **e**) and **f**) and highlights a prominent density in the map derived from the particles in HEPES buffer but not from those in phosphate buffer.

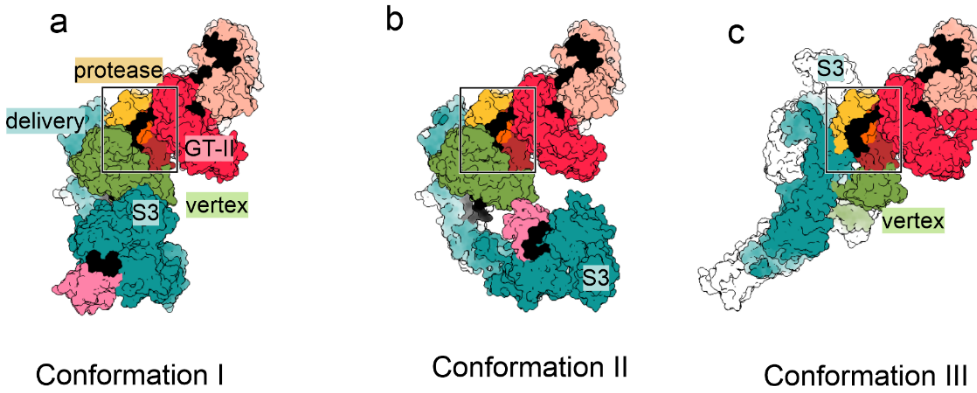

Supplementary Figure 7 | The protease domain is embraced by GT-II, the vertex domain and the delivery domain. a-c) Molecular surface representations of the models of conformations I (**a**), II (**b**) and III (**c**) in the same orientation as the protease domain shown in the main Figure 3b. **b**) and figure 3a show the same surface view. The square indicates the position of the protease domain, which has the same accessibility in conformation I and II but is more concealed by the delivery domain in conformation III. The underlying rearrangements in the delivery domain bring its subdomain S3 in close contact with the protease domain.

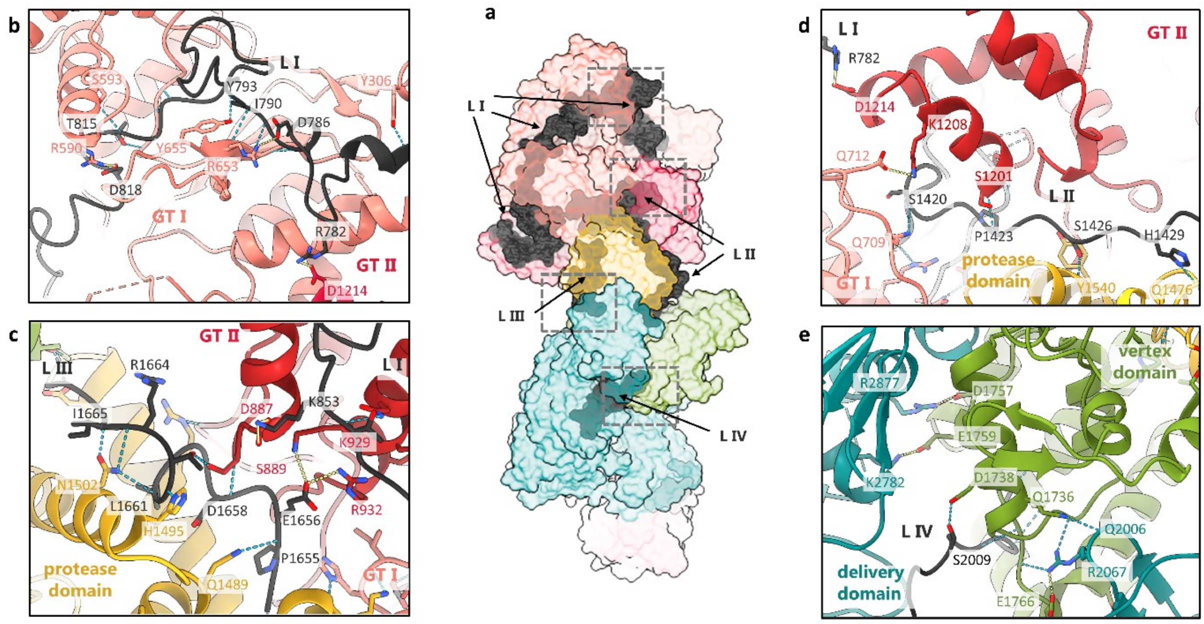

Supplementary Figure 8 | Domain-domain and linker-domain interactions in LifA in conformation I. a, Model of LifA in surface representation with close-ups of the linker regions (**b-e**) indicated by squares in **a**. The close-ups show the model in cartoon representation. The side chains of hydrogen bonded residues are shown together with the label of the residue number and the hydrogen bond as dotted line. The domains are coloured the same as in the main Figure 1. The orientations in the close-ups (**b-e**) differ from the overview in **a**. The linkers are at the interfaces between the domains and interconnect several domains by hydrogen bonds.

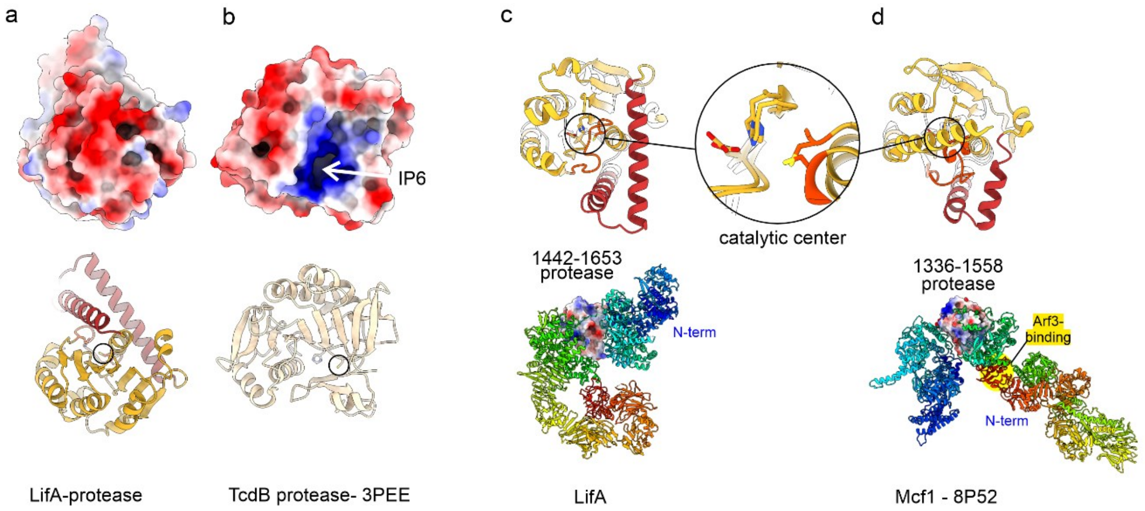

Supplementary Figure 9. | Comparison of the LifA-protease domain with the protease domains of other toxins. **a, b**) Comparison with the protease domain of TcdB (**b**). Top: electrostatic surface representation of the protease domain of LifA (**a**)and the protease domain of TcdB (**b**, pdb: 3PEE [8]) with bound inositol-hexa-kis-phosphate (IP6, not shown). The two protease domains were aligned with matchmaker in ChimeraX. The domains are shown in cartoon representation below their respective electrostatic surface representation. The positions of the catalytic cysteines are outlined with a circle in the cartoon representations. TcdB has a basic flap close to the catalytic cysteine. The protease of TcdB requires binding of an IP6 to this flap for activation. Such a basic flap is absent in the protease domain of LifA (**a**).

c, **d**) Comparison of the protease domains of **c**) LifA and **d**) Mcf1[9]. The protease domain of Lymphostatin (**c**, residues 1442-1653) and of Mcf1 (**d**, residues 1336-1558) were aligned to each other with Chimera X. The top panels show the protease domain with the crossing helices in dark red and the functionally relevant loop in orange. The proteases of both toxins belong to the same C58_PaToxP-like family of proteases. The side chains of the catalytic triad are shown, and the approximate position of the catalytic centre is indicated by a circle. The close-up shows the superposition of both catalytic centres at the position indicated by the circle. The bottom panels of **c**) and **d**) show the full-length proteins of LifA (**c**) and Mcf1 (**d**, 8P52[9]) with the protease domain shown as electrostatic surface representation in the same orientation as the protease domains in the above panels. The residues outside of the protease domain are shown in a ribbon representation and coloured in rainbow from blue to red according to increasing residue numbers. Note the opposite arrangement of the N-terminal arm and the C-terminal arm relative to the protease domain in LifA and Mcf1. The protease of Mcf1 is activated by binding of Arf3[9]. The approximate binding site of Arf3 in Mcf1 is indicated by a yellow background.

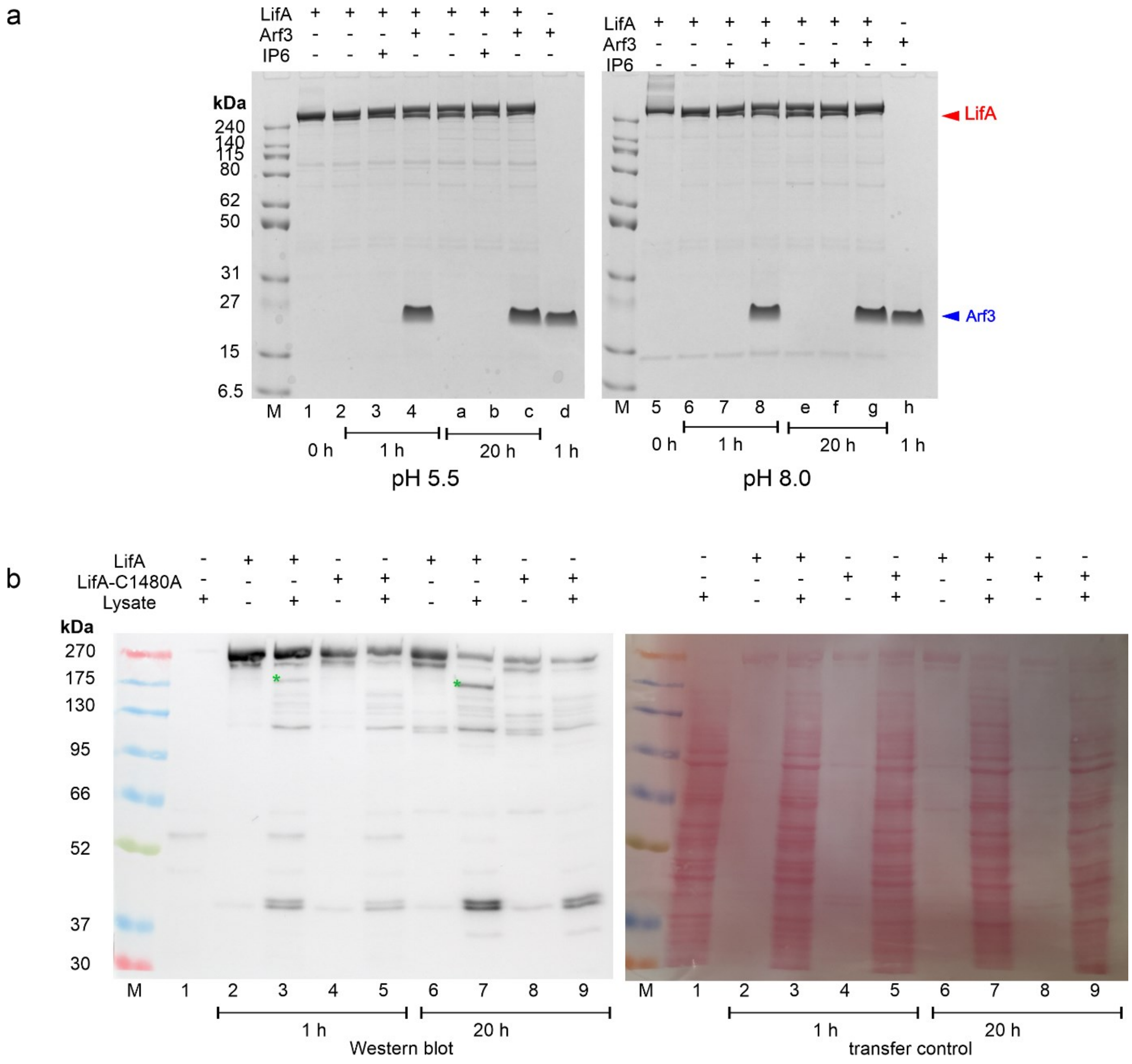

Supplementary Figure 10. | Activation of autocatalytic cleavage of LifA a, Activation of auto-catalytic cleavage of LifA by Arf3 or IP6. The SDS-PAGEs are the same as shown in Figure 3f with the same labelling of the lanes. The complete gels are shown with additional lanes for t=20 hours (lanes a-c and e-g) and purified Arf3 (lanes **d** and **h**). **b**, Activation of auto-catalytic cleavage by HEK-cell lysates tested by Western blot analysis. The Western blot on the left is the same as shown in Figure 3g. The right panel shows the Ponceau S-stained transfer control. At least three independent repeats showed similar results (n=3). Source data are provided as a Source Data file.

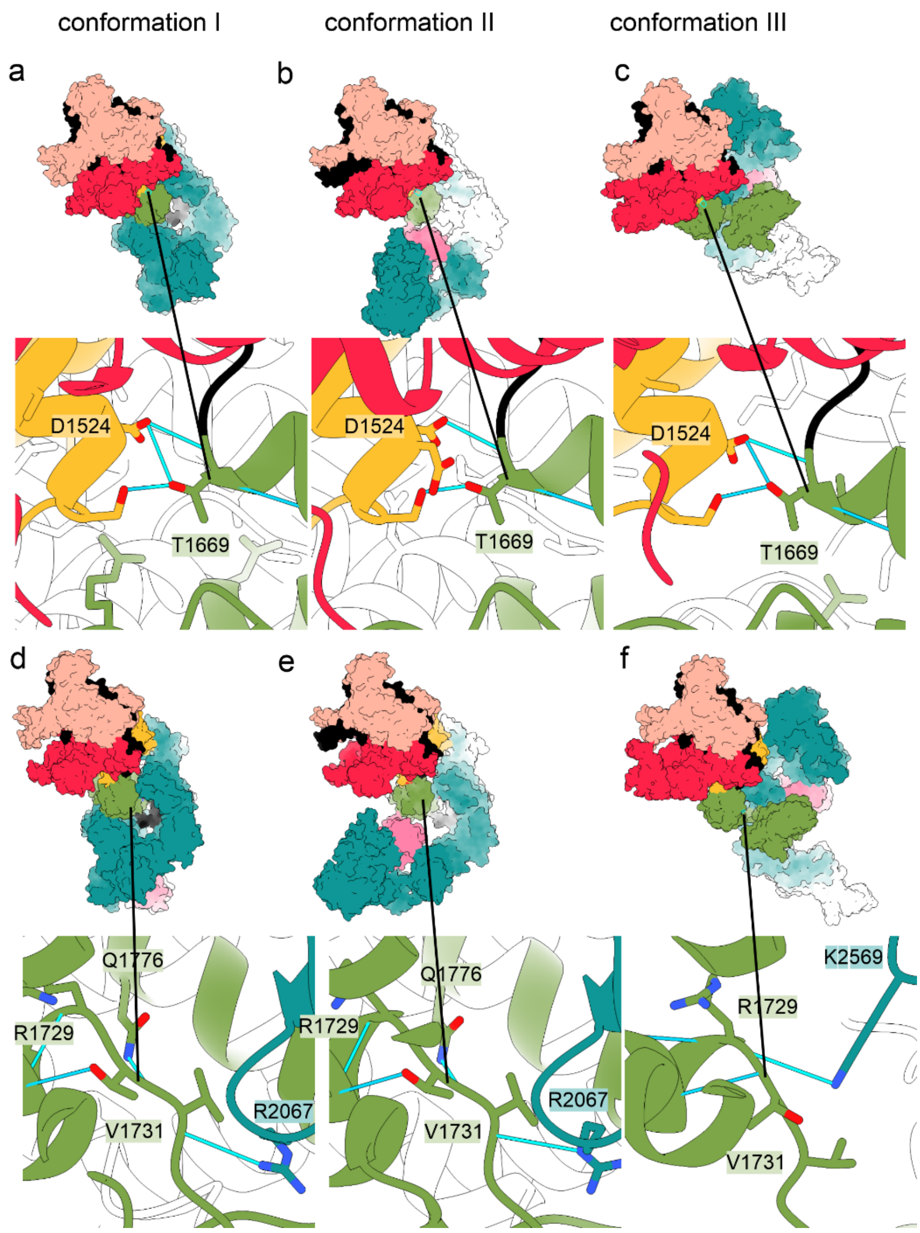

Supplementary Figure 11. | Common and unique hydrogen bonds between the vertex domain and other domains in conformations I, II and III. a-c) The N-terminal subdomain of the vertex forms a hydrogen-bond between T1669 and D1524 at the tip of the crossing helices of the protease domain. This hydrogen bond is common to conformations I, II and III.

d-e) Shows hydrogen-bonds between residues 1729-1730 in the N-terminal subdomain of the vertex and the delivery domain. In conformations I and II this region interacts with R2067 of the S1 subdomain in the delivery domain and with Q1776 in the C-terminal subdomain of the vertex domain. **f**) In conformation III the interaction with the large subdomain of the vertex is lost and a hydrogen-bond is formed with K2569 in S1 of the delivery domain.

The hydrogen-bonds were calculated with Chimera X and are shown as light blue lines. The surface representations of the models of the whole LifA are shown in the same orientation as the closeups underneath. The black line between the representation of the whole LifA model and the closeup connects the same position.

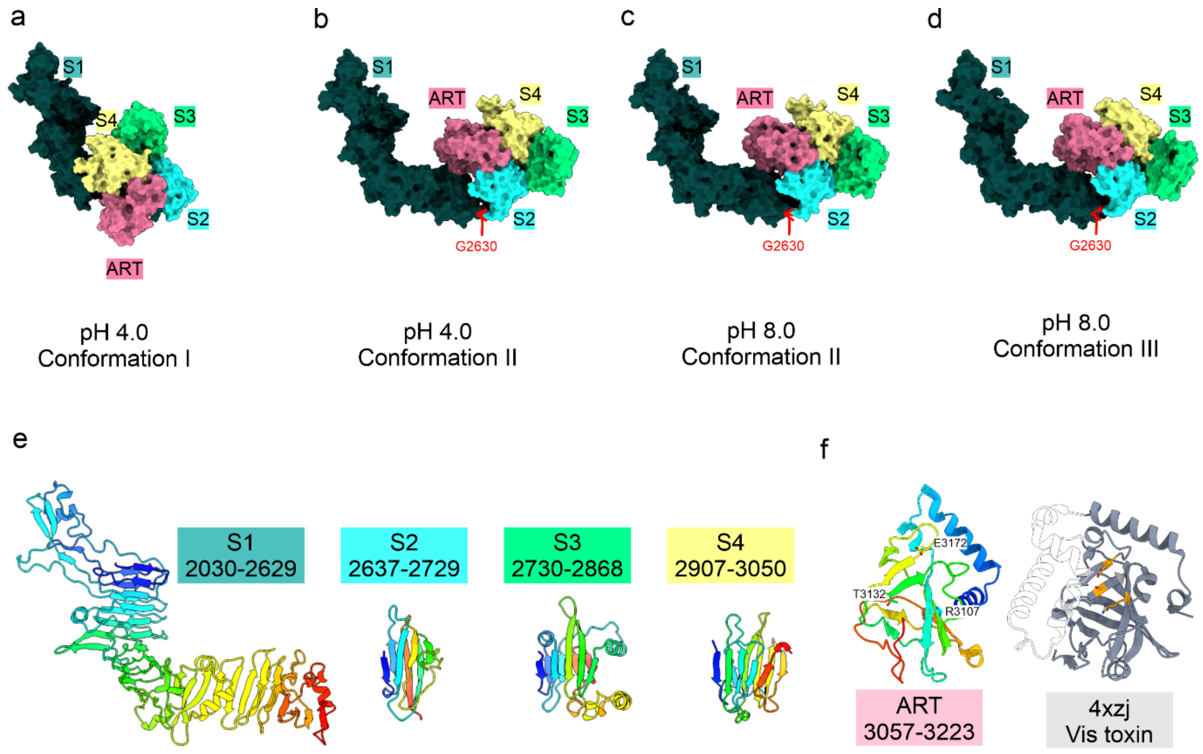

Supplementary Figure 12. | The delivery domain together with the ART-domain change their relative orientations in the different conformations. a-d) Surface representation of the models of the C-terminal arm of LifA in conformations I, II and III. The C-terminal arm consists of the delivery domain and the ART-domain (pink). The delivery domain is subdivided into four subdomains (S1-S4, which are coloured in dark green (S1); cyan (S2); light green (S3) and yellow(S4)). The models in **a-d** are aligned along S1. Conformations II at pH 4 (**b**) and at pH 8 (**c**) and conformation III at pH 8 (**d**) have the same overall arrangement of the subdomains and the ART-domain. Conformation I (**a**) differs from this arrangement by a rigid body rotation of S2-S4 together with the ART-domain around a virtual hinge at G2630 (red arrow).

e) Subdomains S1-S4 are shown in ribbon representation and are coloured in rainbow from their respective N-terminus (blue) to their respective C-terminus (red). The subdomains are shown at the same scale in an arbitrary orientation with the N-terminus close to the upper left side.

f) The ART-domain (left) is shown in the same orientation as the Vis toxin (right, pdb: 4XZJ[10]). which is an ADP-ribosyltransferase. Vis-toxin was identified by FoldSeek[2] as a structural homologue of the ART-domain. Vis-toxin has a characteristic R-S/T-E motif, which is also present in the ART-domain of LifA but shifted. Therefore, the R-T-E residues in the ART-domain of LifA do not form an intact catalytic centre.

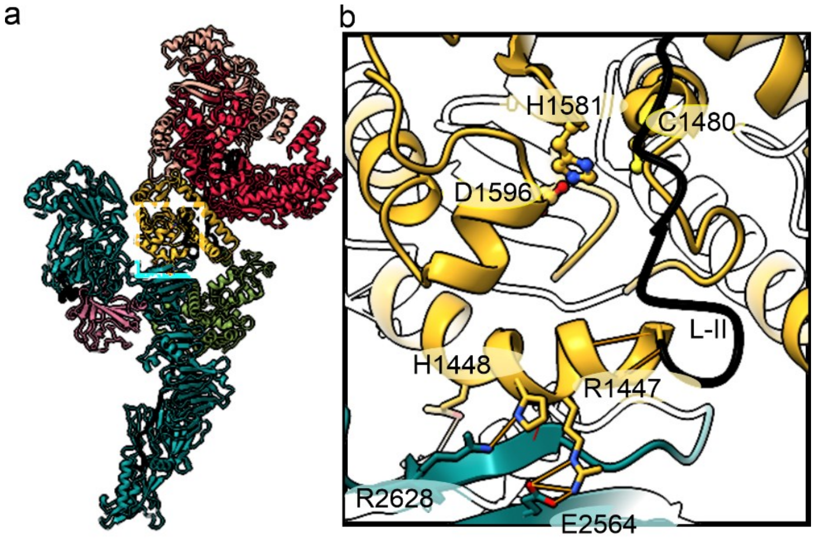

Supplementary Figure 13. | The S1 subdomain of the delivery domain makes several hydrogen bonds with the N-terminal helix of the protease domain in conformation III. a) Model of LifA in conformation III. The square outlines the position of the closeup in **b**). **b**) Closeup of the protease domain (yellow) in conformation III. The view is centred on the catalytic triad (C1480, H581, D1596). L-II is shown in black and is a potential target for autocatalytic cleavage. L-II connects directly to the N-terminal helix of the protease domain, which forms several hydrogen bonds and salt bridges with the C-terminal end of the S1-subdomain of the delivery domain. The interactions are H1448-R2628, R1447-E2564 and Q1451-R2608. These interactions are specific for conformation III and are absent in conformations I and II. This interaction site could support a pulling mechanism in which pulling on the N-terminal helix of the protease domain by S1 would open the catalytic cleft and drag the preceding L-II into the active site of the protease.

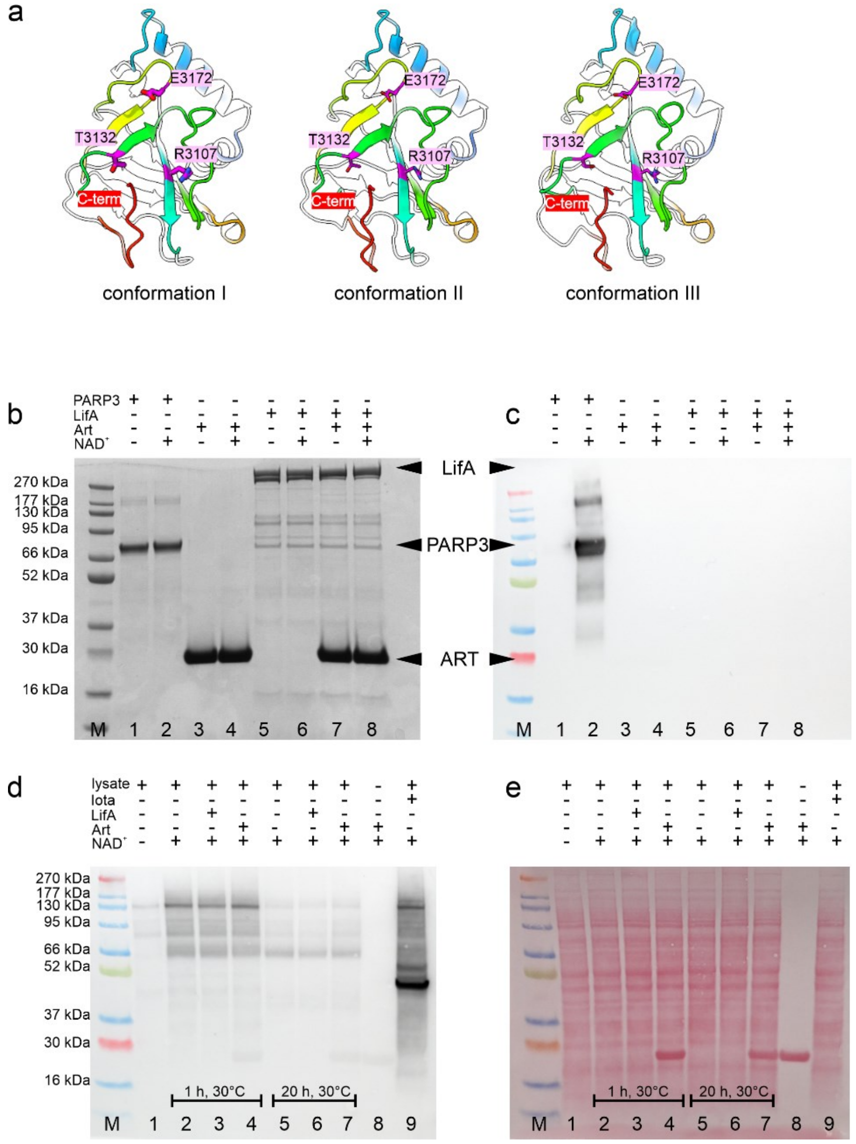

Supplementary Figure 14. | The ART-domain of LifA is inactive. **a**) The ART-domain of conformations I (same as shown in main Figure 5a), conformation II and conformation III are shown side by side with the three catalytic residues R3107, T 3132 and E3172, coloured in magenta. In all three conformations the potential NAD$^+$ binding site is occupied by the C-terminus (red) of LifA. The R-T-E triad does not form a compact catalytic centre suggesting that the ART-domain is inactive in the three conformations. **b, c**) PARP3, LifA and the ART-domain were tested for auto-ADP-ribosylation upon addition of NAD$^+$. The assay composition is indicated above. **b**) shows an SDS-PAGE stained with Coomassie and **c**) the Western blot of a gel ran in parallel under the same conditions. The approximate positions of LifA, PARP3 and the ART-domain are indicated by a black arrowhead. The blot was developed with anti-pan-ADP-ribose binding reagent. Only PARP3, a human mono-ADP-ribosyltransferase, showed NAD$^+$ dependent auto-ribosylation but not LifA or the ART-domain. **d**) shows the same Western blot as in Figure 5b together with **e**) the Ponceau S loading control. This illustrates that the same amounts of HEK cell lysate were loaded in lanes 1-7 and 9. Three independent repeats showed similar results (n=3). Source data are provided as a Source Data file.

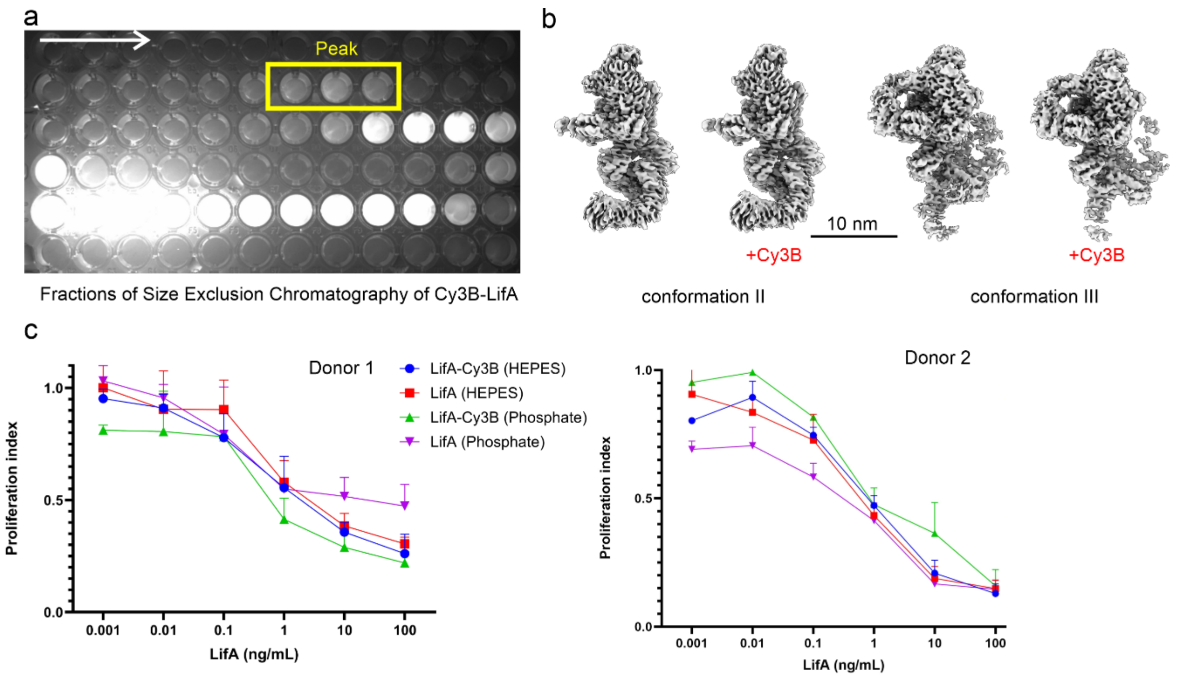

Concentration-dependent inhibition of ConA-stimulated T cells by Cy3B-maleimide-labelled LifA

**Sample:**	**LifA-Cy3B (HEPES)**	**LifA (HEPES)**	**LifA-Cy3B (phosphate)**	**LifA (phosphate)**
Donor 1 ED50 (ng/mL):	0.68± 0.02	0.7± 0.3	0.57± 0.13	0.15± 0.03
Donor 2 ED50 (ng/mL):	0.9± 0.4	0.55± 0.12	0.6± 0.6	1.1± 0.4

Supplementary Figure 15. | LifA-Cy3B is structurally indistinguishable from unlabelled LifA and inhibits mitogen activated T-Lymphocyte proliferation similarly efficient as unlabelled LifA. a) Fluorescence of the fractions from SEC were collected in a 96 well plate and imaged with a gel documentation device in fluorescence mode. The fractions start at the top left corner and the fraction numbers increase from left to right and from top to bottom. The peak fraction containing LifA is outlined in yellow and corresponds to the same fractions marked in Supplementary Figure 2. The SEC separates LifA from free dye and labelled fragments. Only the indicated peak fractions were used for imaging and proliferation assays.

b) Structures of LifA in phosphate buffer at pH 8.0 before and after labelling were determined by cryo-EM. Data of labelled and unlabelled particles were processed together to determine the consensus maps for the subsequent local refinements of conformation II and III. **b**) shows the maps calculated from the subsets of labelled and unlabelled particles at the end of the consensus refinements. Labelled and unlabelled particles contributed similarly to both conformations and yielded comparable results suggesting that labelling had no major impact on the structure of LifA.
c) LifA purified in phosphate buffer or HEPES buffer, before and after labelling was tested in its potency to inhibit mitogen activated T-Lymphocyte proliferation in two healthy donor cell populations. LifA and LifA-Cy3B were similarly potent in inhibiting proliferation with ED50 values ranging between 0.15 and 1.1 ng /ml. The buffer composition during purification (HEPES vs phosphate buffer) had no effect on the ED50. Source data are provided as a Source Data file.

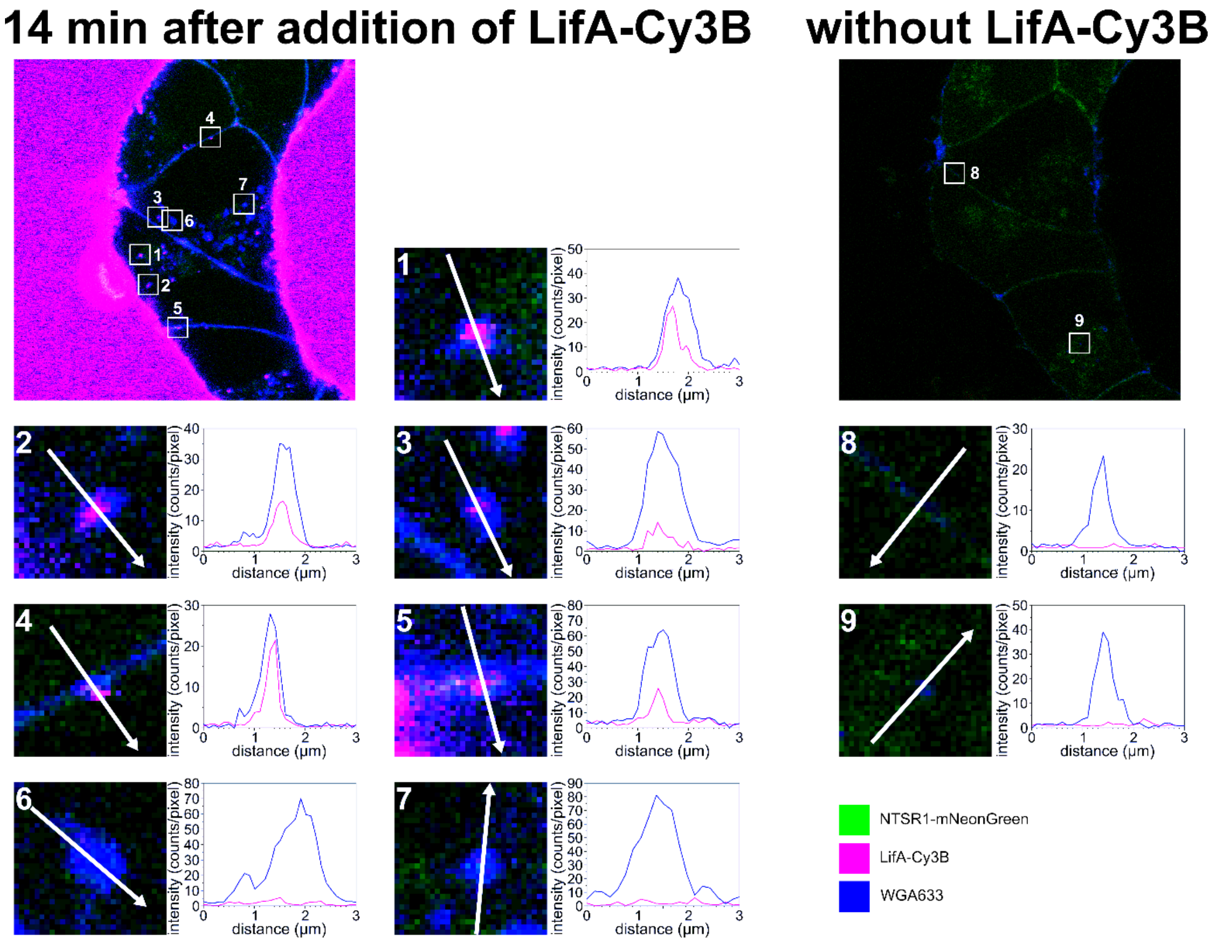

Supplementary Figure 16. | Fluorescence microscopy of Cy3B-labelled LifA at the plasma membrane and inside living HEK293T cells at 37°C. The confocal three-colour images of HEK-293T cells correspond to the main Figure 7, i.e., before (right panels) and 14 min after addition of LifA-Cy3B (left panels). NTSR1-mNeonGreen (false-coloured green) was excited with a pulsed laser at 488 nm and fluorescence was detected in the spectral range between 500 and 550 nm. LifA-Cy3B fluorescence (magenta) was excited with 561 nm and detected between 580 and 630 nm. WGA633 (blue) was excited with 640 nm and detected between 650 and 720 nm. Line accumulation mode was chosen for each excitation laser using the Abberior Expertline FLIM-FRET microscope. Enlarged 3 x 3 μm^2 areas in the numbered sub-panels were selected spots on the plasma membrane as well as spots inside the cells. Intensity profiles were obtained with Imspector (Abberior Instruments) and plotted across each sub-panel as shown by white arrows. In the presence of LifA-Cy3B in the extracellular growth medium, spots 1 to 3 and related profiles showed internalised LifA-Cy3B clusters which were co-localised with WGA633. Spots 4 and 5 depicted LifA-Cy3B clusters on the WGA633-stained plasma membrane. Spots 6 and 7 showed endocytosed WGA633 with negligible LifA-Cy3B fluorescence, or remaining background in the spectral range, respectively. Before addition of LifA-Cy3B, spots 8 (membrane) and 9 (inside the cell) showed WGA633 fluorescence but no fluorescence signal in the spectral channel for LifA-Cy3B, as expected. We note that the background was subtracted for each channel separately. Intensities are given as photon counts per pixels in the profile plots, and colour intensity contrast was enhanced in the images for better visibility. Source data are provided as a Source Data file. LifA-Cy3B was discriminated from fast autofluorescence by the 2.7 ns fluorescence lifetime (data not shown).

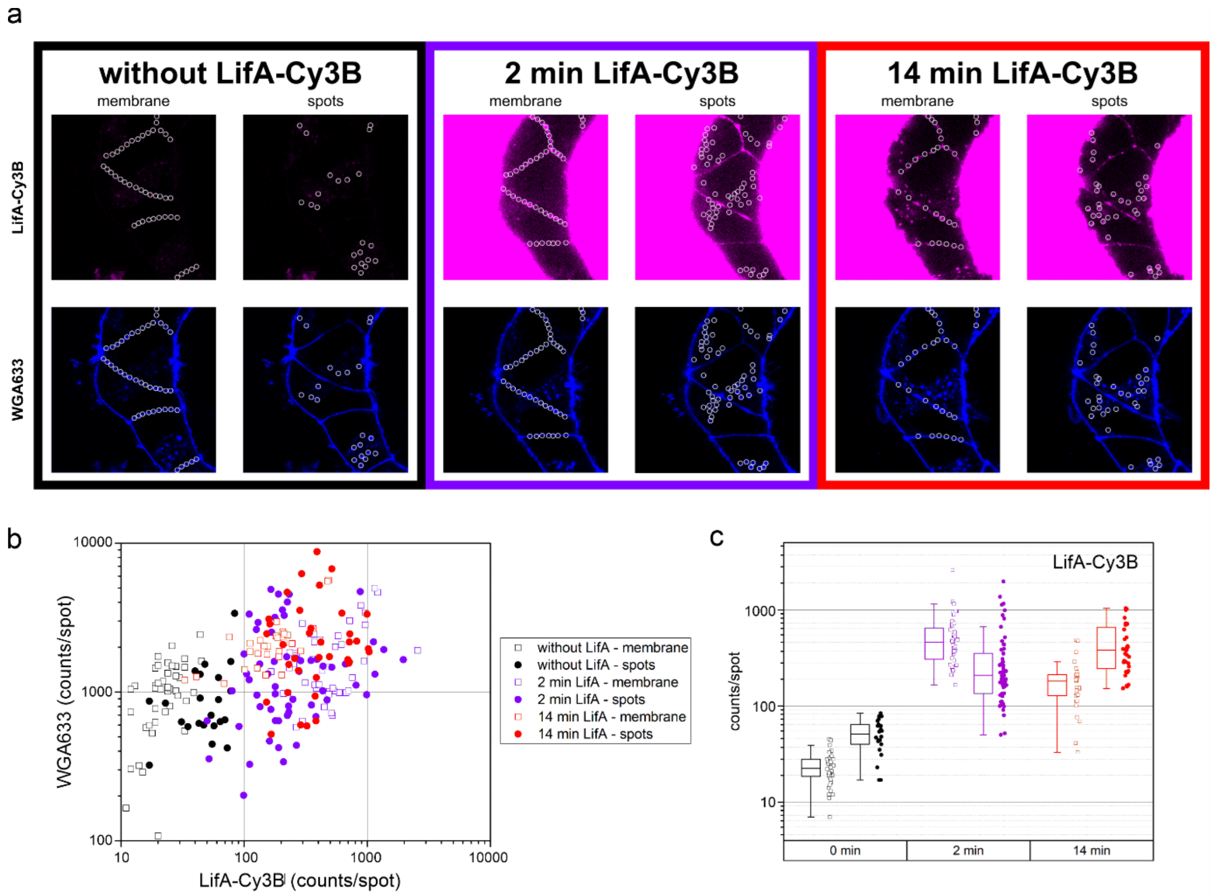

Supplementary Figure 17. | Brightness analysis for LifA-Cy3B uptake by living HEK293T cells stained with WGA633. a) Positions of the selected fluorescence spots on the plasma membranes at cell-cell interfaces and within the cells (see Figure 7). Left, before addition of LifA-Cy3B; middle, 2 min after addition; right, 14 min after addition. Cells were pre-stained with WGA633 for 15 min. Spot brigthness was analysed with a custom python script (Python Software Foundation). Positions were manually selected. Afterwards the photon counts per channel were added up inside a circle with 7 pixel radius. **b**) 2D scatter plot of spot brightnesses on the membrane (empty squares) and within the cells (filled dots), before addition of LifA-Cy3B (black), 2 min after addition (purple) and 14 min after addition of LifA-Cy3B (red). **c**) box-with-whiskers plots for the LifA-Cy3B spectral channel for the same data as in **b**) with median (black line within the boxes) and the 25% / 75% bounds for the boxes. Uptake of LifA-Cy3B into the HEK cells was characterised by a significant increase in photon counts per spot exceeding the upper autofluorescence limit (< 100 counts/spot, black filled circles at time “0 min”) with a mean of 51 counts/spot. After 2 min, the 44 selected internal spots showed a mean brightness in the LifA-Cy3B spectral channel of 335 counts/spot with a standard deviation (s.d.) of 335 counts/spot (purple filled dots). The very broad standard deviation indicated that some internal spots did not contain a LifA-Cy3B molecule. After 14 min, the 36 selected internal spots showed a mean brightness of 434±258 counts/spot (±s.d.) (red filled dots). All selected spots contained LifA-Cy3B, but the spot brightness distribution was still very broad. This small increase in mean internal spot brightness from 2 to 14 min was considered “not significant”. For LifA-Cy3B found on the membranes at the cell-cell interfaces, the mean brightness was 554±403 counts/spot (±s.d.) 2 min after addition (purple empty squares). After 14 min, the mean brightness was lowered to 186±108 counts/spot (±s.d.) potentially caused by photo-bleaching of LifA-Cy3B which were immobilised on the membrane (red empty squares). Direct excitation of WGA633 with 561 nm yielded the residual mean brightness on the membrane of 23±8 counts/spot (±s.d.) (black empty squares). Source data are provided as a Source Data file. Excitation of WGA633 with 640 nm yielded similar mean brightnesses for membranes and

for internalised spots, with a slight increase from 1000 at “0 min” to 2200 counts/spot at “14 min” (data not shown).

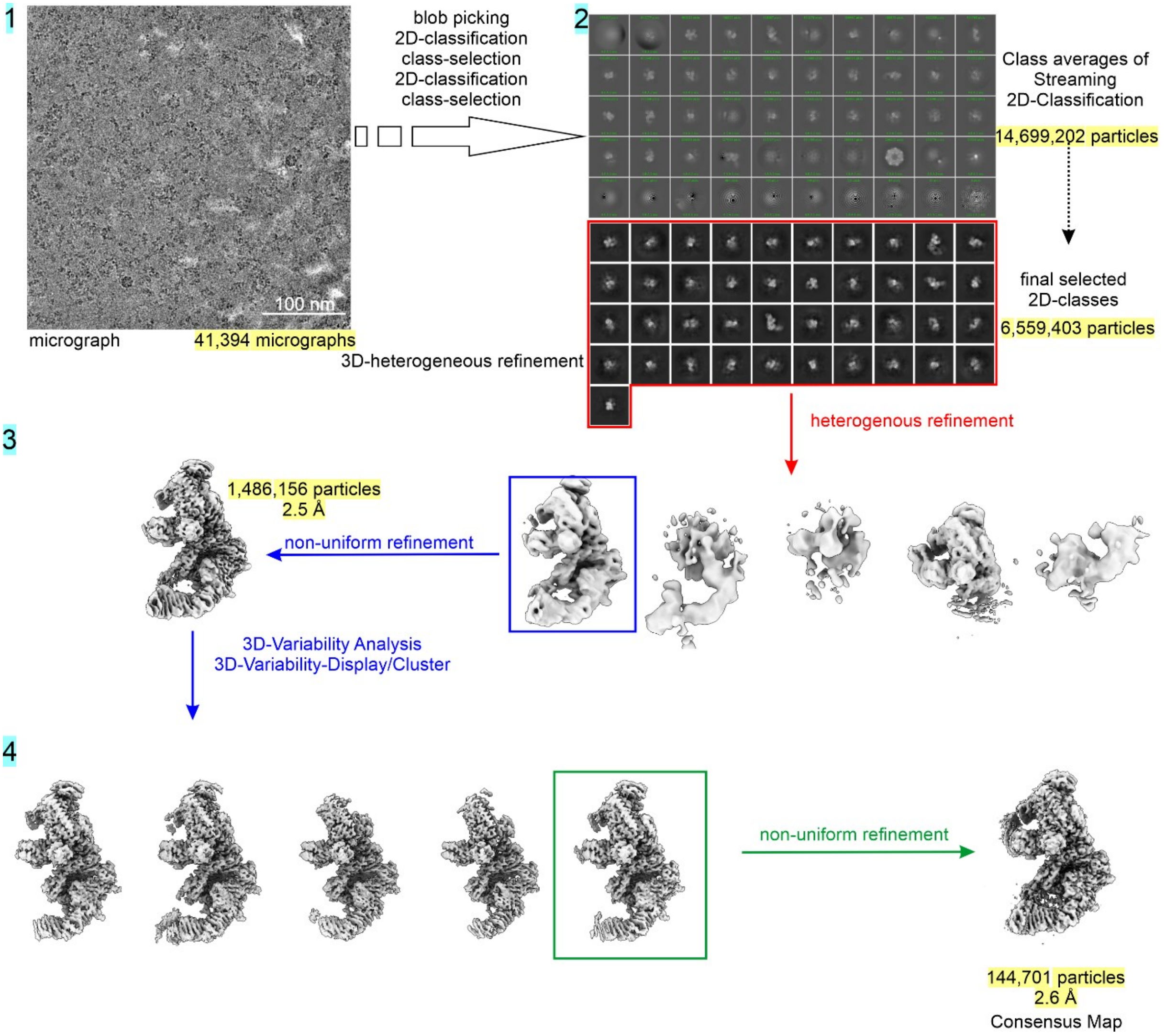

Supplementary Figure 18. | Workflow of processing of LifA at pH 4 in phosphate buffer-starting with blob-picked particles in CryoSPARC[11]. Cyan numbers highlight the milestones of the processing. 1) 41,394 micrographs of LifA were motion-corrected, and dose weighted during a live session in CryoSPARC. Particles were "blob picked" during the live session. In total, 14,699,202 particles were extracted. 2) The extracted particles were 2D-classified in streaming as part of the live session (streaming 2D-classification). 2D-Classes were selected based on the quality and appearance of their averages followed by another round of 2D-classification and selection. The final, selected classes are shown and contain 6,559,403 particles. 3) The selected particles were classified in a heterogeneous 3D-refinement. The refinement was primed with 5 references from processing of small subsets during the life session. Reference 1 represented the whole particle, reference 2 was a small fragment, which resembled the delivery domain and reference 3 was a small fragment that resembled the GT-domain. References 2 and 3 were aligned on the map of the whole particle. References 4 and 5 were copies of references 2 and 3 but mass centred. At the end of the heterogeneous refinement, all 5 classes were populated. The first class (1,486,156 particles, blue square) and the fourth class (2,291,713 particles) reached 4.8 Å resolution, which was Nyquist frequency of this refinement. The first class (blue outline) represented the whole LifA-molecule. The particles were reextracted in full size and refined with non-uniform refinement. The final map had a resolution of 2.5 Å with a cFAR of 0.19. 4) The refined particles were subjected to 3D-Variability analysis followed by clustering into 5 clusters with 3D-Variability display. Each cluster was separately refined with non-uniform refinement. The cluster indicated by the green square had 144,701 particles and reached a resolution of 2.6 Å in the subsequent non-uniform refinement. Compared to the other clusters, this was the highest resolution and was most isotropic with a cFAR of 0.43. The map of the cluster was used for generating templates in the

subsequent template-based selection of particles (Supplementary Figure 19). The map also served as consensus map for generating a combined map of conformation I from locally refined parts with Phenix.

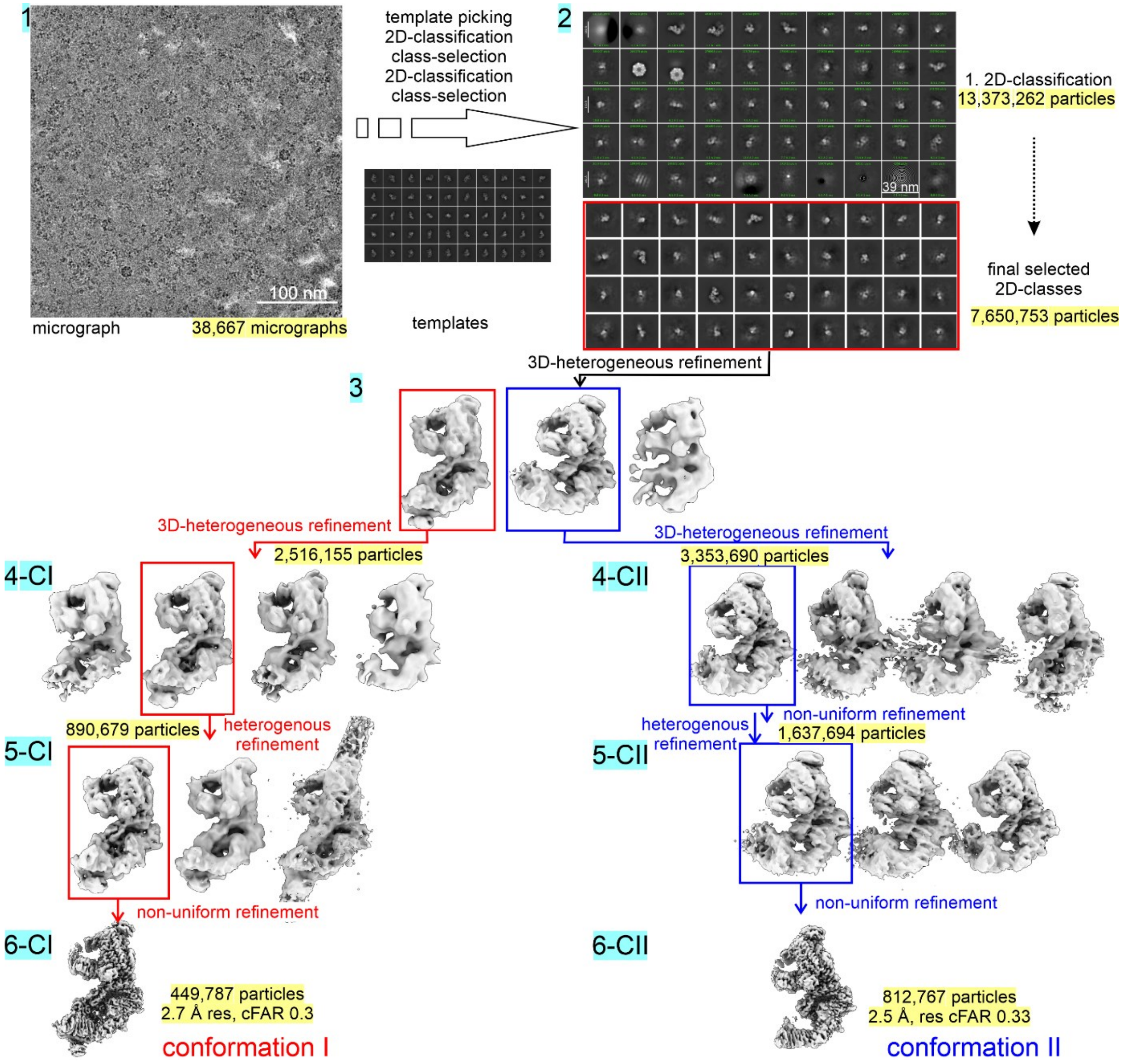

Supplementary Figure 19. | Workflow of processing of LifA at pH 4 in phosphate buffer - Separating conformations I and II in CryoSPARC[11]. Cyan numbers highlight the milestones of the processing. 1) 38,867 of the motion corrected, and dose weighted movies were selected for further processing based on a CTF-Fit resolution of < 4 Å and a defocus between 200 nm and 2000 nm. The particles were template picked, using 50 projections calculated from the consensus map of the blob picked particles at pH 4 (Supplementary Figure 18). A total of 13,373,262 particles were template picked and extracted. 2) The extracted particles were 2D-classfied into 50 classes (upper panel). 2D-Classes were selected based on the quality and appearance of their averages followed by another round of 2D-classification and selection. The final, selected classes are shown and contain 7,650,753 particles. 3) The selected particles were classified in a heterogeneous 3D-refinement. The refinement was primed with 3 references from the previous processing of smaller subsets of this data set. Reference 1 represented conformation I, reference 2 represented conformation II and reference 3 was the consensus map of the blob-picked particles (Supplementary Figure 18). At the end of the heterogeneous refinement, one class (2,516,155 particles, red outline) resembled conformation I another class (3,353,690 particles, blue outline) resembled conformation II and the third class (no outline) contained mainly empty picks. The particles from conformation I (red branch, CI) and from conformation II (blue branch, CII) were processed separately following a similar strategy. 4) Selected particles were separated by another round of heterogeneous refinement into 4 classes each. The respective references were three copies of the class average of the particles from the same branch from the previous refinement and

one copy of the class average from the other class. The best class was identified based on visual inspection either of the class averages or the non-uniform refined class. The selected class is outlined and contains 890,679 particles for conformation I and 1,637,694 particles for conformation II. 5) The selected classes were heterogeneously refined into 3 classes. 6) Each of the classes was non-uniformly refined and the classes with the most consistent densities and the highest cFAR in the orientation diagnostic were selected (as outlined). 6-CI) At the end of the CryoSPARC processing, the map of conformation I had a resolution of 2.7 Å and a cFAR of 0.3. The map included 449,787 particles 6-CII) The none-uniform refinement of the 812,767 particles in conformation-II gave a map with a resolution of 2.5 Å and a cFAR of 0.33. The particles from each branch were exported with pyem[12] and further processed with RELION5.0[13].

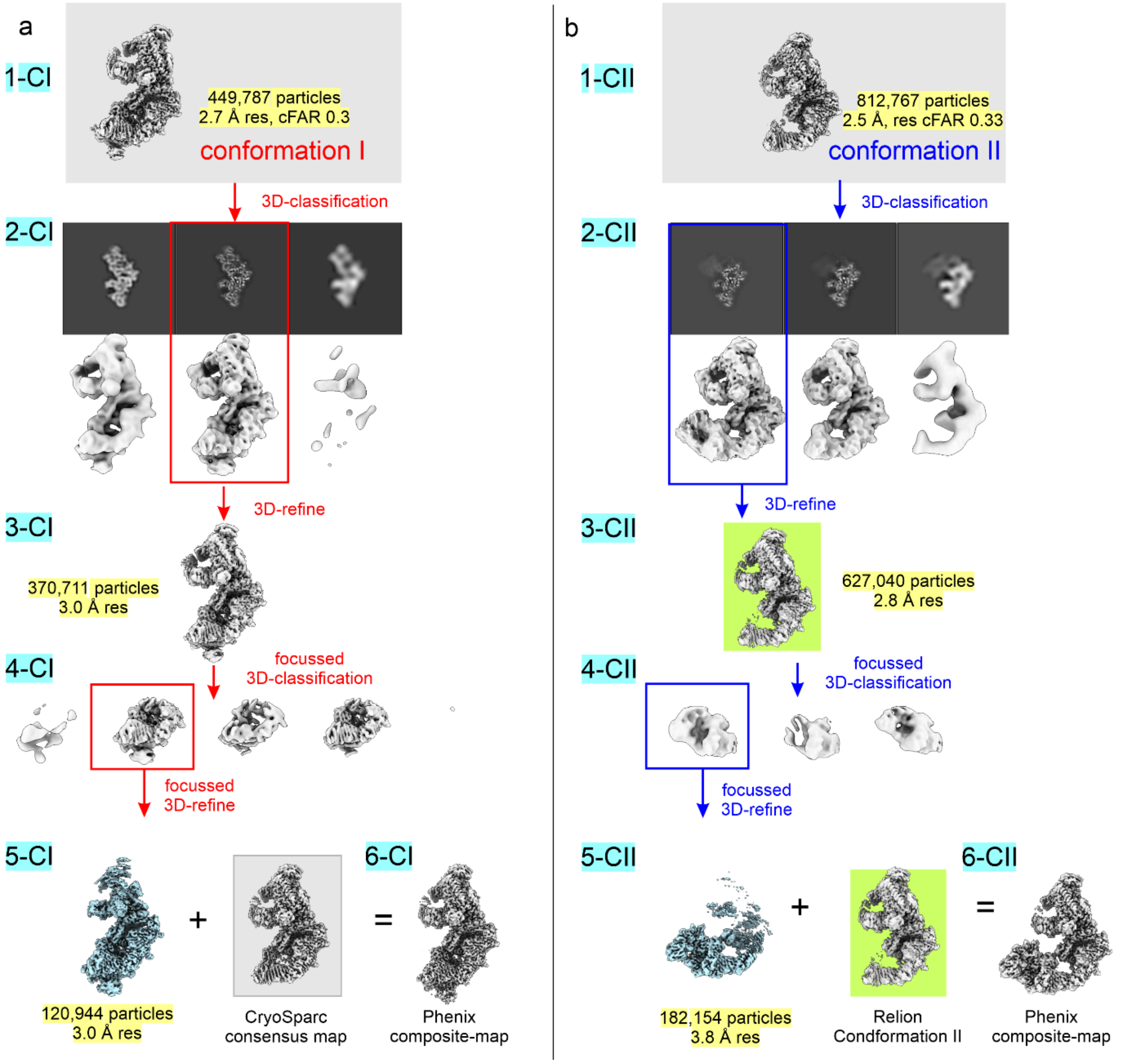

Supplementary Figure 20. | Workflow of LifA at pH 4 in phosphate buffer – Polishing conformations I and II in RELION[13]. Cyan numbers highlight the milestones of the processing. Numbers with yellow background refer to the number of particles and the nominal resolution. **a**) polishing of conformation I and **b**) polishing of conformation II. 1) Polishing starts with particles of the final none-uniform refinement of conformations I and II in CryoSPARC (grey background Supplementary Figure 19). The metadata of the refinements were converted with pyem [12] to star format. The star files were imported to RELION 5 2) The imported particles were classified into 3 classes without alignment. 3) The best class (outlined) was selected and 3D-refined using local refinements. 4) After 3D-refinement the particles were 3D-classfied without alignment using a spherical mask that was focussed on the C-terminal tip. 5) The best class (outline) subjected to local 3D-refinement focussed on the C-terminal tip. 6) The C-terminal parts after focussed refinement were combined with a consensus map of the whole LifA to a composite map using Phenix[14] ("combine focussed maps"). Surface representations of the composite maps are shown. These maps were used for real-space-refinement with Phenix. The consensus map for conformation I was the consensus map (grey background) at the end of the CryoSPARC processing of the blob-picked particles (Supplementary Figure 18). The consensus map for conformation II (green background) was the map of the 3D-refinement in 3-CII (green background).

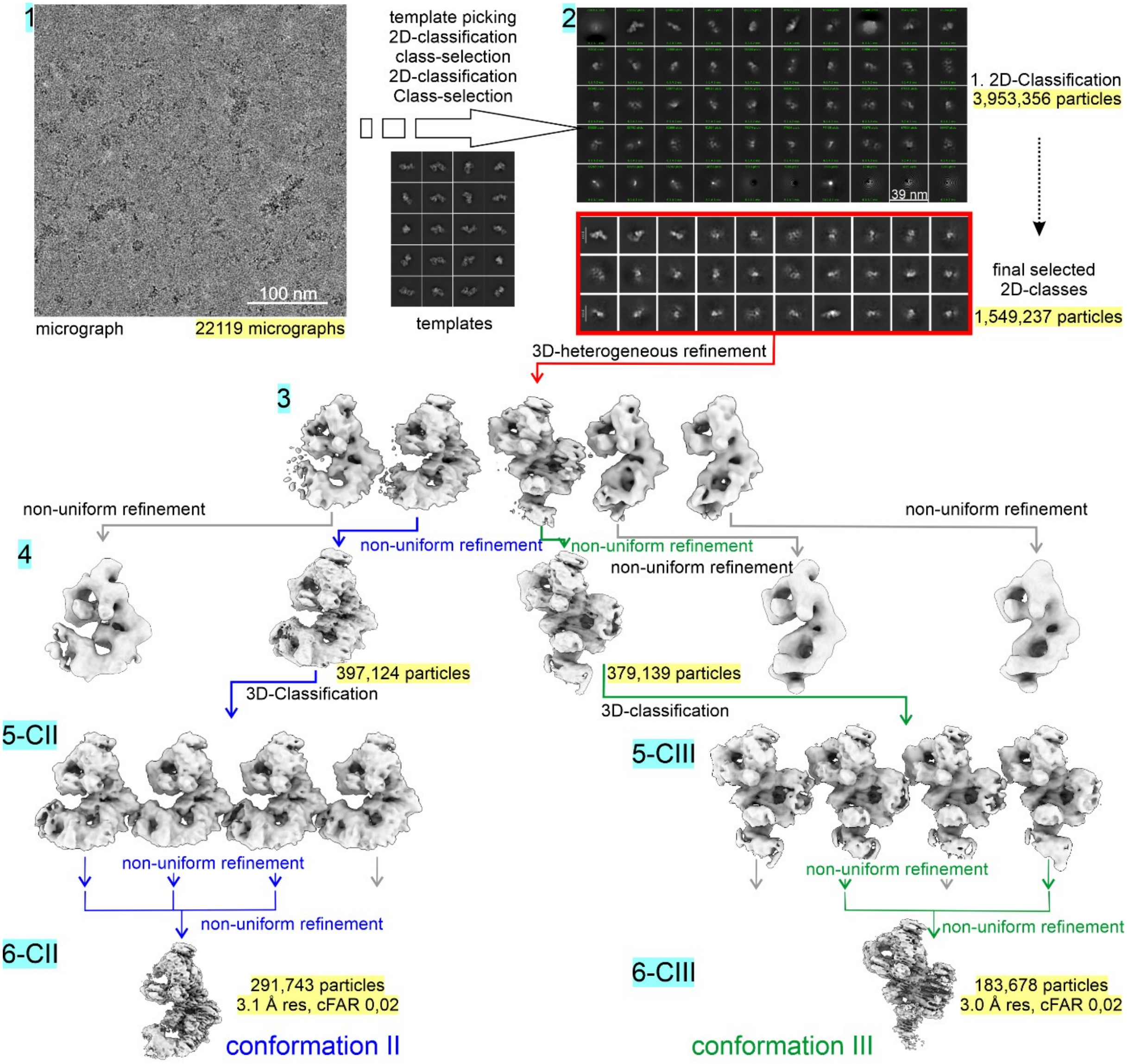

Supplementary Figure 21. | Workflow of processing of LifA at pH 8 in phosphate buffer. Separating conformation II and III in CryoSPARC[11]. Cyan numbers highlight the milestones of the processing. 1) 22119 micrographs of LifA were motion corrected, and dose weighted during a live session in CryoSPARC. Particles were template picked, using 20 projections calculated from the final map of conformation I at pH 4 (Supplementary Figure 19). The templates were low pass filtered to 20 Å resolution. The bias towards conformation I was intended to verify whether conformation I was absent. The 3,953,356 particles were 2D-classified in streaming during the live session. 2) 2D-Classes were selected based on the quality and appearance of their averages followed by another round of 2D-classification and selection. The final, selected classes are shown and contain 1,549,237 particles. The selection was generous to not loose rare views. 3) The selected particles were classified in a heterogeneous 3D-refinement. The refinement was primed with 5 references from previous processing of other data sets. Reference 1 and 2 were the same and derived from conformation II at pH 4.0. Reference 3 was derived from conformation III from a preliminary analysis of a partial data set and references 4 and 5 were the same and derived from conformation I at pH 4.0. At the end of the refinement, all 5 classes were populated but only two classes (blue and green follow-on arrows) reached a resolution of 6 Å, which was the Nyquist frequency of this heterogeneous refinement. 4) Each class from the heterogeneous refinement was refined separately with non-uniform refinement. Only 2 of the refinements gave

interpretable maps and reached higher resolution. One derived from class 1 and presented conformation II (blue branch) and the other derived from class 2 and represented conformation III (green branch). Notably, the maps of class 0, 3 and 4 also had lower grey values than the other two maps suggesting that these classes represented mainly empty picks. The particles in conformation II (blue branch, CII) and conformation III (green branch, CIII) were processed separately following a similar strategy. 5) The particles were subjected to 3D-classification without alignment into 4 classes. This was followed by an individual non-uniform refinement of each class (not shown). 6) The particles from the best non-uniform refinements were combined and re-refined in the final non-uniform refinement. At the end of this processing 297,743 particles grouped to conformation II (6-CII) and 183,678 particles grouped to conformation III (6-CIII). Both maps where anisotropic in resolution as indicated by the low cFAR of 0.02. Particles from each branch were exported with pyem[12] and further processed with RELION5.0[13].

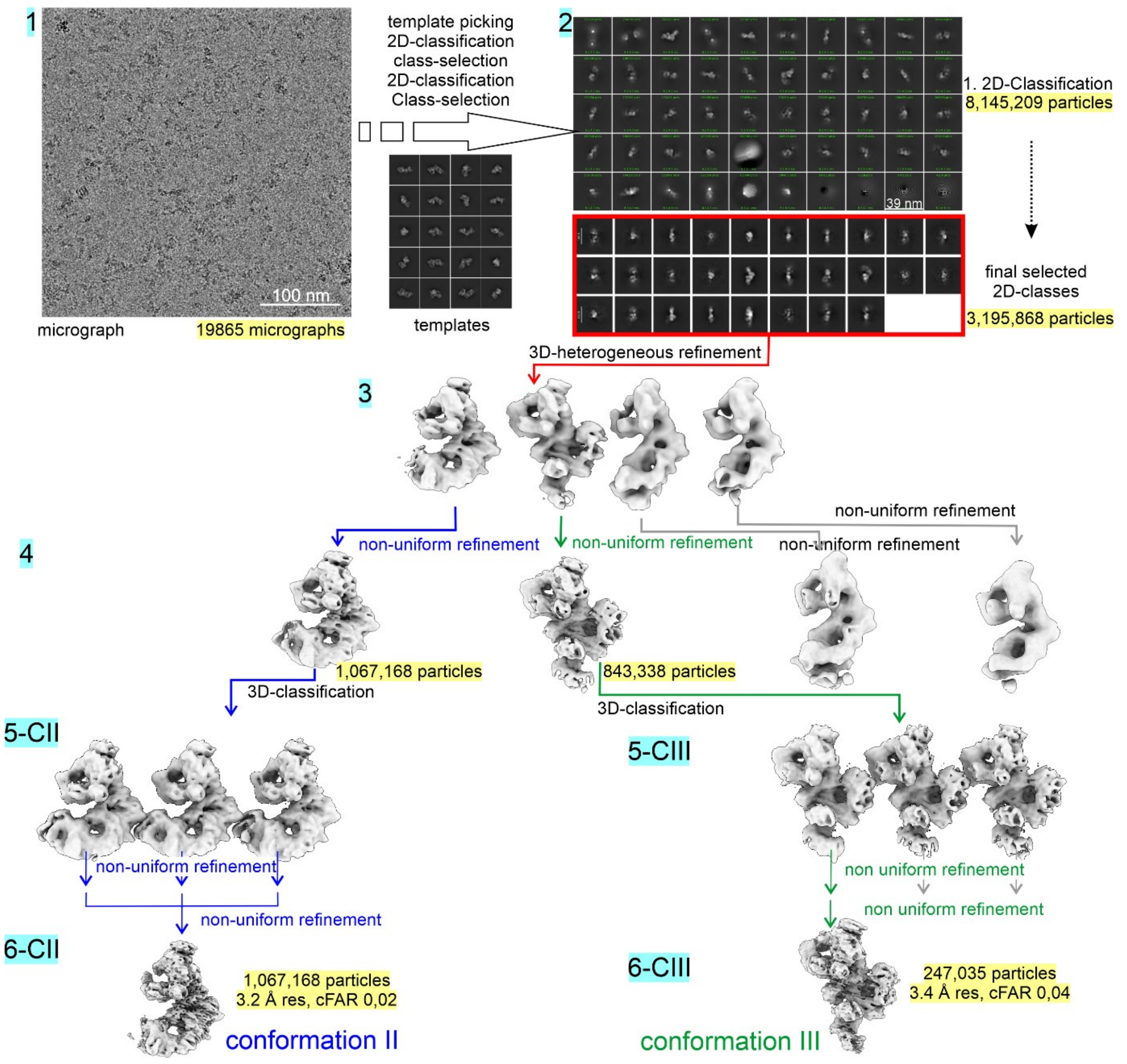

Supplementary Figure 22. | Workflow of processing of LifA-Cy3B at pH 8 in phosphate buffer. Separating conformation II and III in CryoSPARC[11]. Cyan numbers highlight the milestones of the processing. 1) 19865 micrographs of LifA were motion corrected, and dose weighted during a live session in CryoSPARC. Particles were template picked, using 20 projections calculated from the final map of conformation I at pH 4 (Supplementary Figure 19). The templates were low pass filtered to 20 Å resolution. The bias was intended to verify whether conformation I was absent. The 8,145,209 particles were 2D-classified in streaming during the live session. 2) 2D-Classes were selected based on the quality and appearance of their averages followed by another round of 2D-classification and selection. The final, selected classes are shown and contain 3,195,868 particles. 3) The selected particles were classified in a heterogeneous 3D-refinement. The refinement was primed with 4 references from previous processing of other data sets. Reference 1 was derived from conformation II at pH 4.0. Reference 2 was derived from conformation III from a preliminary analysis of a partial data set and references 3 and 4 were identical and were derived from conformation I at pH 4.0. At the end of the heterogeneous refinement, all 4 classes were populated but only two classes (blue and green follow-on arrows) reached the resolution of the Nyquist frequency of this heterogeneous refinement. 4) Each class from the heterogeneous refinement was refined separately with non-uniform refinement. Only 2 of the refinements gave interpretable maps and reached higher resolution. One derived from class 0 and presented conformation II (blue branch) and the other derived from class 1 and represented conformation III (green branch). Notably, class 2 and 3 also had lower grey values than the other two classes suggesting that

these classes represented mainly empty picks. The particles in conformation II (blue branch, CII) and conformation III (green branch, CIII) were processed separately following a similar strategy. 5) The particles were subjected to 3D-classification without alignment into 3 classes. This was followed by an individual non-uniform refinement of each class (not shown). 6) The particles from the best non-uniform refinements were combined and re-refined in the final non-uniform refinement. At the end of this processing 1,067,168 particles grouped to conformation II (6-CII) and 247,035 particles grouped to conformation III (6-CIII). Both maps where anisotropic in resolution as indicated by the low cFAR of 0.02 for conformation II and 0.04 for conformation III. Particles from each branch were exported with pyem[12] and further processed with RELION5.0[13].

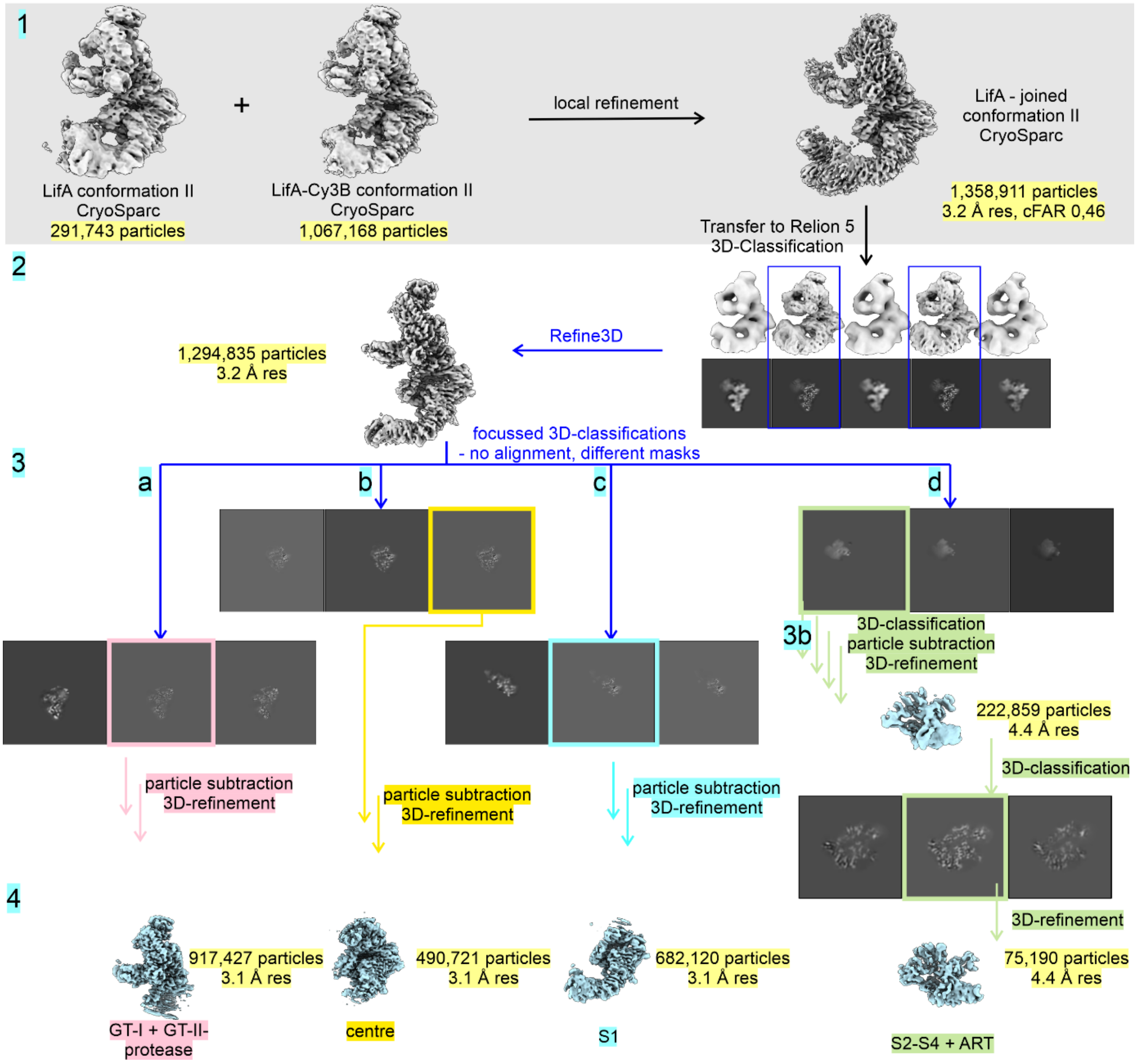

Supplementary Figure 23. | Joined RELION[13]-processing of conformation II of LifA and LifA-Cy3B at pH 8 in phosphate buffer. Cyan numbers highlight the milestones of the processing. The final steps of joining the data in CryoSPARC are indicated by a grey background. 1) The sub-sets of LifA in conformation II (291,743 particles, Supplementary Figure 21) and of LifA-Cy3B (1,067,168 particles, Supplementary Figure 22) were joint in a local, consensus refinement in CryoSPARC[11]. The particles were restacked and exported with pyem [12] to RELION 5 [13]. 2) The imported particles were classified without alignment into 5 classes with blush regularization. The classes are presented as surface presentation calculated with ChimeraX[3] and by a central slice through the map below. Both presentations are not at the same scale. Most particles grouped into classes 2 and 4, which are outlined by a blue square. These classes were selected (1,294,835 particles) and subjected to a local 3D-refinement, which gave a resolution of 3.2 Å. This map served as consensus map for generating the combined map from the locally refined parts in Phenix. 3) Masks, representing the a) GT-I, GT-II and protease domain, b) the centre, c) subdomain S1 of the delivery domain and d) subdomains S2-S4+ART were generated. For each mask a 3D-classification of the aligned consensus data into 3 classes was determined. The central slice of the respective classification is shown below. In 3a-c) The class indicated by the square was selected. The selection was based on visual inspection selecting for the most consistent density.4) The classes identified in 3a-3c were subtracted for the background outside of the mask, centred and transferred into a smaller box. The background subtracted particles were locally refined with Refine3D and blush-regularization. The maps of the subtracted particles are shown below

indicating the number of particles included in the map and the resolution. The refinement in branch 3d (S2-S4+ART) involved a multi-step classification and refinement approach as this part is more mobile. The first classification into 3 classes was followed by background subtraction and recentring of the best class. The background subtracted 222.859 particles were 3D-refined giving a resolution of 4.4 Å. As the density looked still inconsistent, we did another round of classification together with a local alignment. The best class contained 75,190 particles and was 3D-refined, giving a resolution of 4.4 Å Comparing this map with the previous map (above) showed much more consistent density despite of having the same resolution. This observation highlights that "resolution" and "population" alone are insufficient guides for identifying a homogeneous subset.

We combined the maps of the branches a-d) with Phenix[14] into a composite map using the map in 2) as the consensus map.

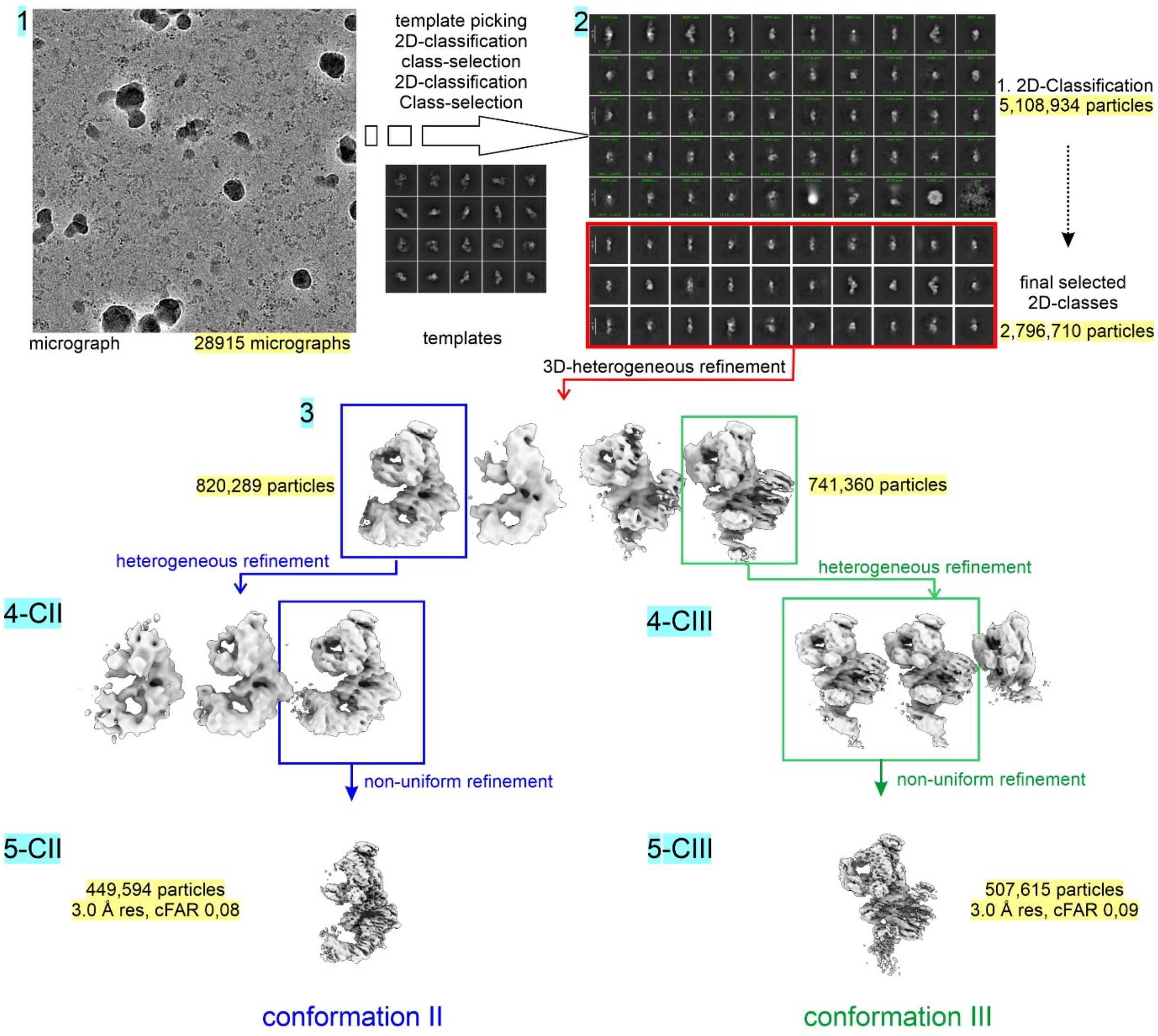

Supplementary Figure 24. | LifA at pH 8.0 (HEPES-buffer): Separating conformation II and III in CryoSPARC[11]. Cyan numbers highlight the milestones of the processing. 1) 28,915 micrographs of LifA were motion corrected, and dose weighted during a live session in CryoSPARC. The particles were template picked, with equally spaced projections of conformation II (20 projections) and III (20 projections). In total, 5,108,934 particle locations were extracted. 2) The particle images were 2D-classified followed by selection of 2D-classes based on the quality and appearance of their class averages. This was followed by another round of 2D-classification and selection. The averages of the final, selected classes are shown and represented 2,796,710 particles. 3) The selected particle images were classified in a heterogeneous 3D-refinement. The refinement was started with 4 references of which two represented conformation II and two represented conformation III. At the end of the heterogeneous refinement all 4 classes were populated but only two classes (blue and green follow-on arrows) reached the resolution of the Nyquist frequency in the heterogeneous refinement. 4) One of these two classes presented conformation II (blue branch) and the other conformation III (green branch). The particles in conformation II (blue branch, CII) and conformation III (green branch, CIII) were processed separately following a similar strategy. 4) In each branch, the selected particles were subjected to another heterogeneous refinement into 3 classes. This identified one class for conformation II (blue outline) and 2 classes for conformation III (green outline) as the subsets that produced the most consistent 3D-maps. 5)

These classes were subjected to another non-uniform refinement. In conformation II 449,594 particles were in the final class and produced a map with 3.0 Å resolution (cFAR=0.08). For conformation III 507,615 particles were averaged in the final map reaching a resolution of 3.0 Å with a cFAR of 0.09. The particles from each branch were exported with pyem[12] for further processing with RELION5.0[13].

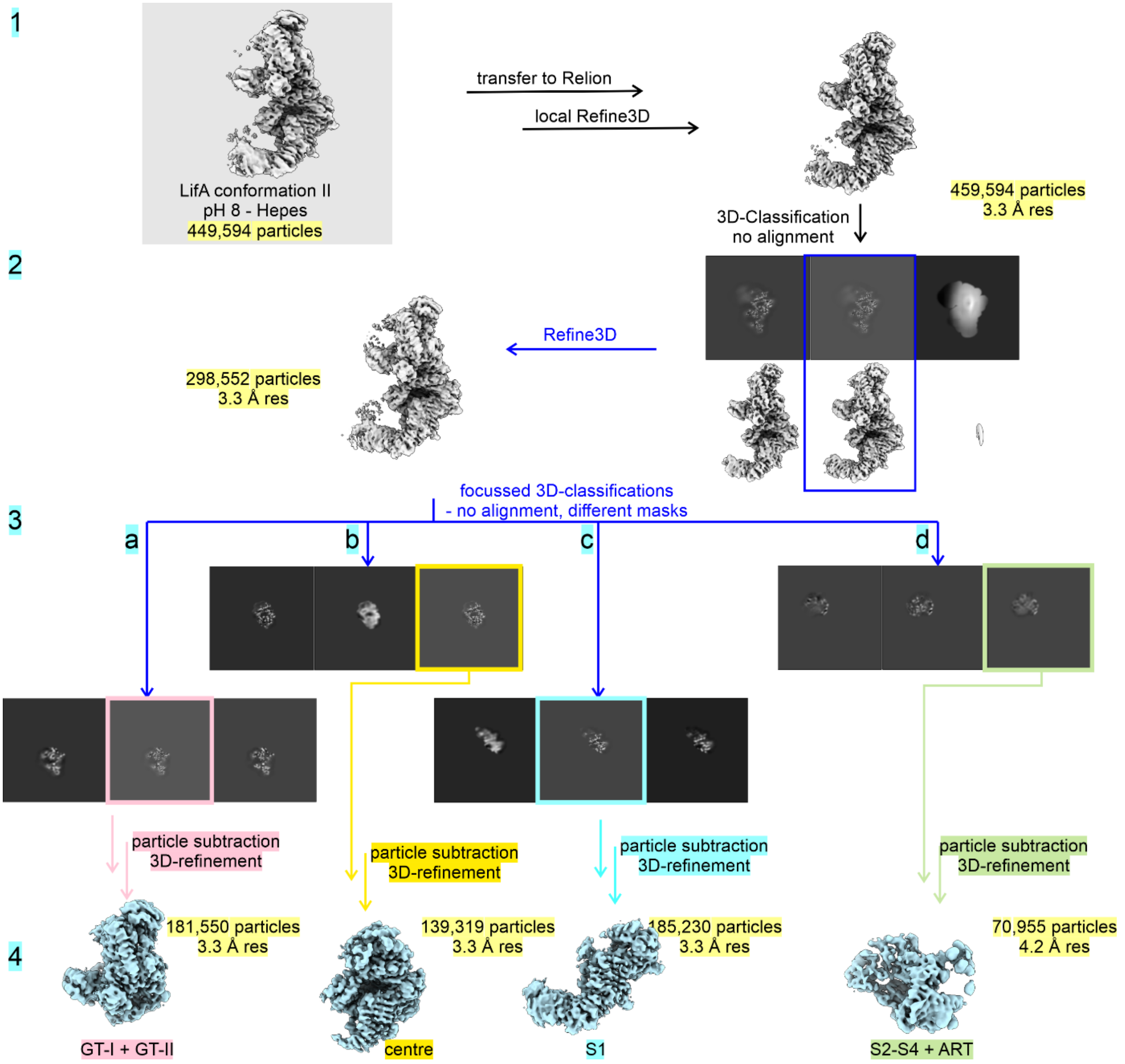

Supplementary Figure 25. | RELION[13]-processing of conformation II of LifA at pH 8 in HEPES buffer. Cyan numbers indicate the milestones of the processing. The grey background highlights the 3D-refinement of conformation II at the end of the CryoSPARC-processing (Supplementary Figure 24). 1) The meta-data of the sub-set was converted with pyem[12] to a star file and imported to RELION 5[13]. The imported 359,594 particles were 3D-refined with local searches using blush regularization. The map at the end of the refinement had a resolution of 3.3 Å. 2) The aligned particles were classified without alignment into 3 classes with blush regularization. The classes are presented as surface presentation calculated with ChimeraX and by a central slice through the map below. Both presentations are not at the same scale. Most particles grouped into classes 2 (blue outline). This class was selected (298,552 particles) and subjected to a local 3D-refinement, which gave a resolution of 3.3 Å. This map served as consensus map for generating the combined map from the locally refined parts in Phenix[14]. 3) Masks, representing the a) GT-I, GT-II domain, b) the centre, c) subdomain S1 of the delivery domain and d) subdomains S2-S4+ART were generated. For each mask a 3D-classification of the aligned consensus map into 3 classes was determined. The central slice of the respective classification is shown below. The class indicated by the square was selected. The selection was based on visual inspection selecting for the most consistent density.4) The selected lasses were subtracted for the background outside of the mask and recentred on the mask and boxed into a smaller box. The background subtracted particles were locally refined with Refine3D and using blush-regularization. The maps of the subtracted

particles are shown together with the number of particles included in the map and the resolution.

The focussed maps of the branches a-d) were combined with the consensus map in 2 to a composite map using Phenix.

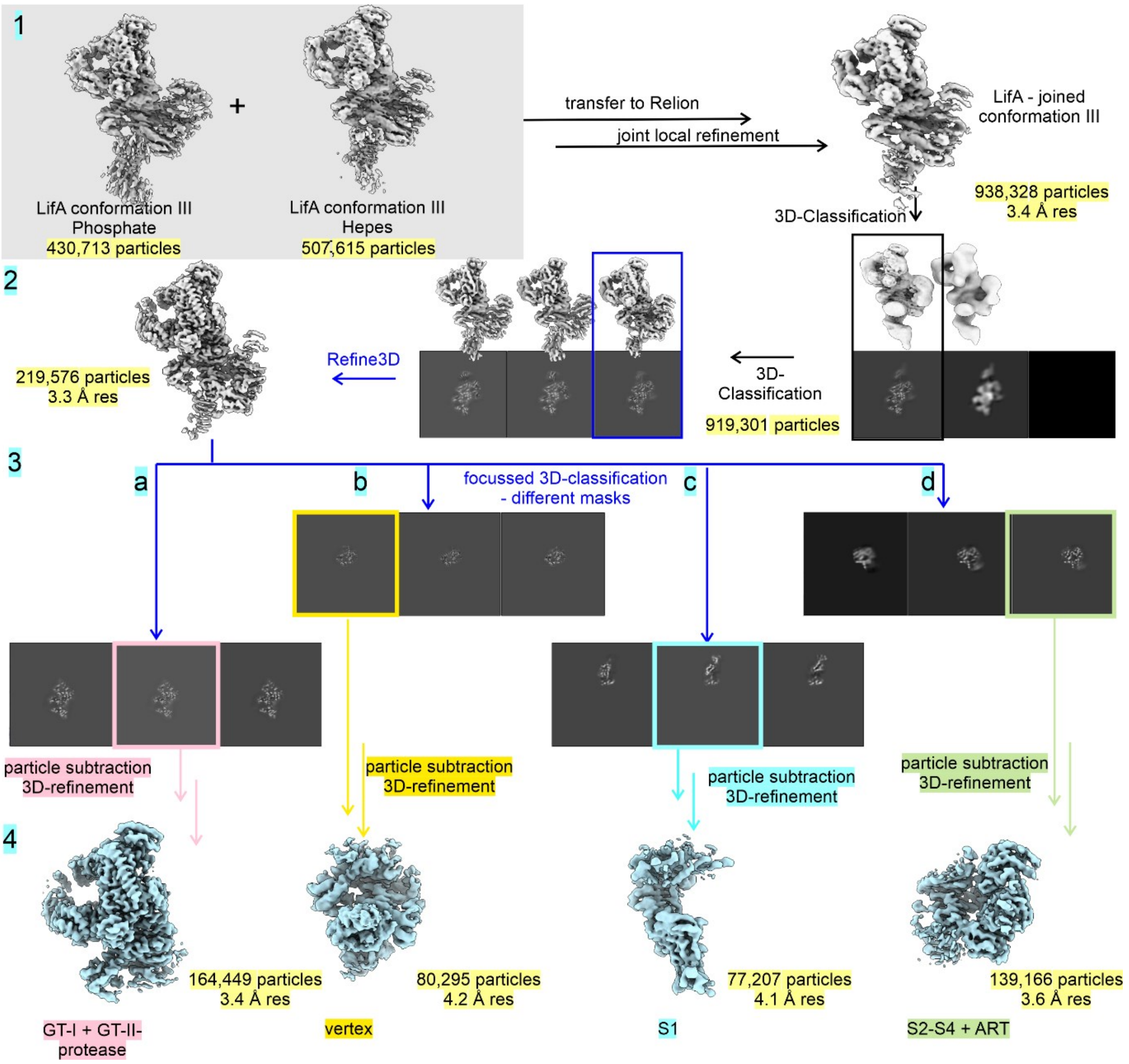

Supplementary Figure 26. | Joined RELION[13] processing of conformation III of LifA at pH 8 in phosphate buffer and in HEPES buffer. Cyan numbers highlight the milestones of the processing. The grey background shows the reconstructions of conformation III in phosphate buffer and in HEPES buffer at the end of separating conformation II and III in CryoSPARC. 1) Both sets of particles were imported to RELION and joined. The joined particle set (938,328 particles) were 3D-refined with local alignments to a common reference. The nominal resolution at the end of refinement was 3.4 Å. 2) The particles were classified without alignment into 3 classes with blush regularization. The class averages are presented as surface presentations calculated with ChimeraX. In addition, class averages are shown as a central slice through the map. Most particles grouped into class 1, which is outlined by a black square. This class was selected (919,301 particles) for further processing and was subjected to another round of 3D-classification but this time with T=8. After 20 iterations without alignment, local alignment was switched on for 5 iterations. All classes were populated with class 3 giving the highest resolution and having the smallest number of particles (indicated by blue square). The 219,576 particles of this class were locally refined and reached a nominal resolution of 3.3 Å. This map was later used as consensus map for generating a composite map with Phenix from the focussed refinements of the sub-maps. 3) Masks, representing the a) GT-I, GT-II and protease domain, b) the vertex with the adjacent protease domain and the S1-subdomain, c) the subdomain S1 of the delivery domain and d) subdomains S2-S4+ART were generated. For each mask a 3D-classification of the aligned particles from the consensus refinement into 3 classes was determined. The central slice of the respective classification is

shown below. In 3a-d) The class selected for further processing is indicated by a square. The selection was based on visual inspection choosing the class with the most consistent density. 4) The particles in the classes marked in 3a-3d were subtracted for the background outside of the mask. The subtracted particles were re-boxed into a smaller box (192 px, 182 Å) and centred on their respective mask. The background subtracted particles were locally refined with Refine3D and blush-regularization. The surface representations present the final maps focussed on their respective mask. For each focussed map the number of particles included in the map and the nominal resolution are given.

The focussed maps were combined into a composite map with Phenix[14]. The map at the end of the processing in 2) was used as consensus map for aligning the focussed maps.

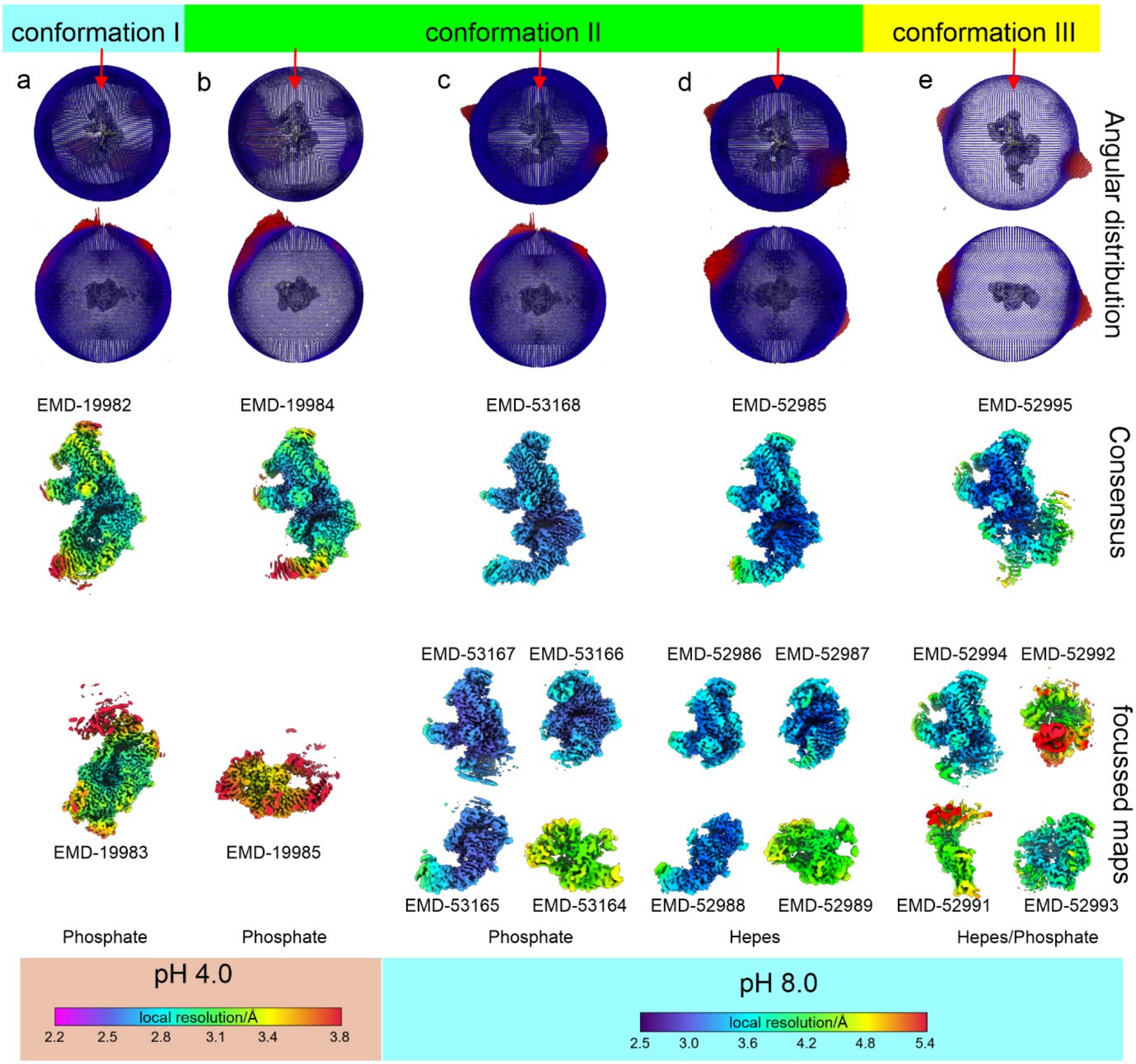

Supplementary Figure 27. | Angular distribution and local resolution of deposited maps. a) Conformation I in phosphate buffer at pH 4.0: b) conformation II in phosphate buffer at pH 4.0, c) conformation II in phosphate buffer at pH 8.0; d) Conformation II in HEPES buffer at pH 8.0; e) conformation III at pH 8.0

The upper two rows show two perpendicular views of the angular distribution of the respective consensus maps below. The red arrows indicate the viewing direction of the angular distribution in the panel below. The angular sampling is indicated by radial cylinders. The length of the cylinders refers to the relative number of views in the respective orientation. Similarly, blue indicates fewer particles in an orientation while red indicates more particles in certain orientations than expected for an even distribution.

The third row shows the consensus maps coloured with the local resolution. This map is shown in the same orientation as the angular distribution in the panel above. Below the maps from the focussed refinements are shown. For each map the EMDB accession code is given. All maps of LifA at pH 4.0 are shown with the same colour code for local resolution. The look-up table is shown below and ranges from magenta (2.2 Å) to red (3.8 Å). The maps of LifA at pH8.0 are also coloured according to local resolution but use a different look-up table (shown below). The colours range from dark blue (2.5 Å) to red (5.4Å). The different look-up tables reflect the different resolution ranges of the maps at pH 4.0 and pH 8.0.

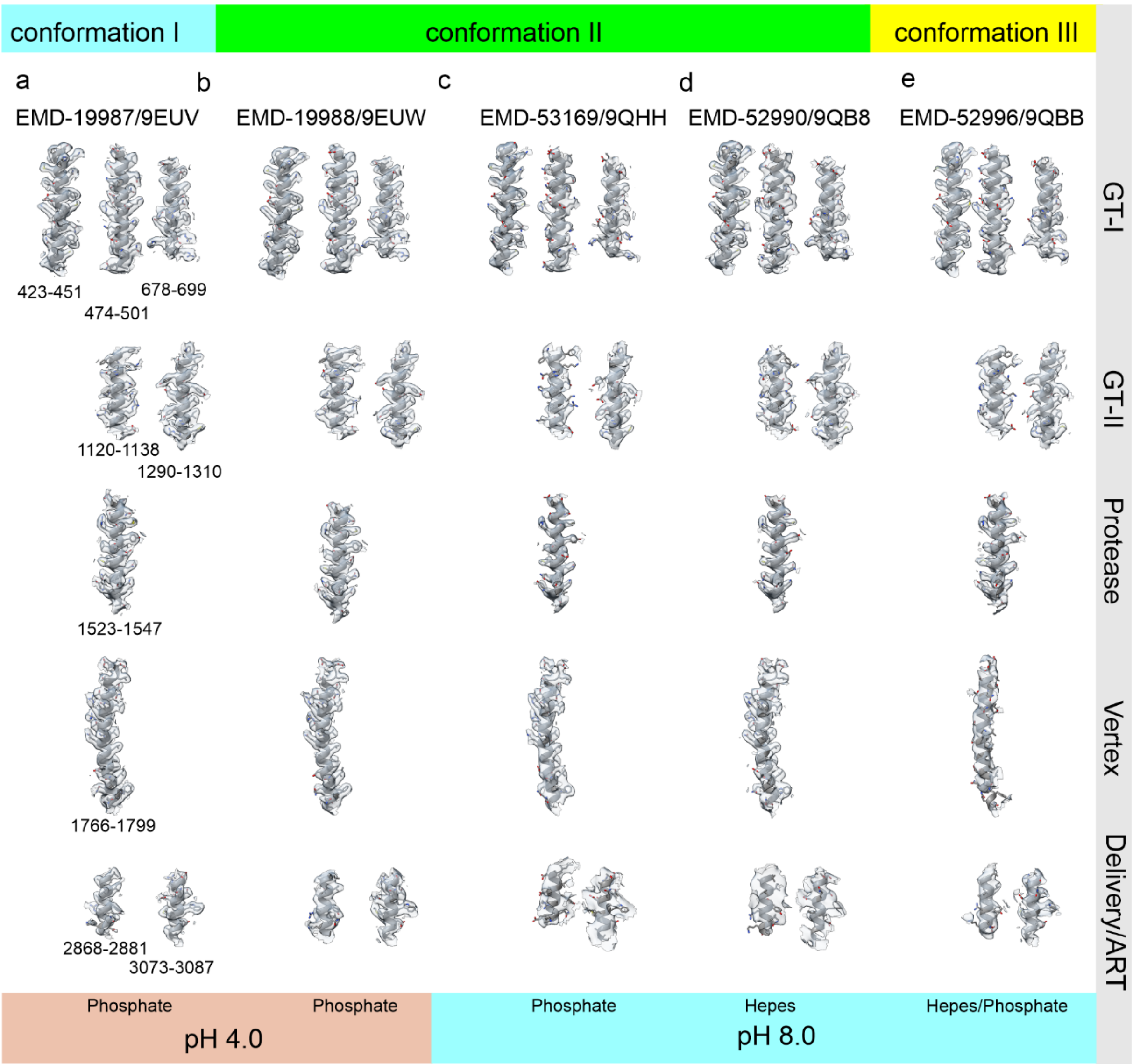

Supplementary Figure 28. | Presentations of composite maps and models for selected helices. a) Conformation I in phosphate buffer at pH 4.0; b) conformation II in phosphate buffer at pH 4.0; c) conformation II in phosphate buffer at pH 8.0; d) conformation II in HEPES buffer at pH 8.0; e) conformation III at pH 8.0

The access codes for the respective composites map in the EMDB together with the access codes of the model in the pdb are listed at the top. The helices are oriented with the N-terminus pointing upwards. The range of residues is shown below the respective helix in a) (conformation I at pH 4.0). The same helices are also shown for the other maps and models in the same row. At least one helix is shown for each domain. In GT-I the residues 423-451 form the longest helix. This helix has no counterpart in GT-II. The other two depicted helices (residues 474-501 and 678-699) have counterparts in GT-II (residues 1120-1138 and 1290-1310). The resolvability of the side chain density for some helices varies in the different maps. However, at least one of the five maps showed side chain density that allowed modelling.

Supplementary Table 1 | Parameters of image acquisition of LifA at different conditions. Each acquisition is shown in a separate column. Data joined in the processing is indicated below (orange) and the processing and model-building is summarized in Supplementary Table 2-7 as indicated.

**Sample**	**LifA pH 4.0 Phosphate**	**LifA pH 6.5 Phosphate**	**LifA pH 8.0 Phosphate**	**LifA-Cy3B pH 8.0/ Phosphate**	**LifA pH8.0 HEPES**
Electron microscope	Krios G3 with X-FEG (Thermo Fisher), Cs=2.7 mm, HT=300 kV				
Camera	Falcon IVi	Falcon III	Falcon IVi	Falcon IVi	Falcon IVi
Camera mode	counting	linear	counting	counting	counting
Energy filter	Selectris (5eV slit)	none	Selectris (5eV slit)		
Movie format	EER	MRC, 40 fractions	EER		
C2/Spot Size	70 µm / Spot 5, nanoprobe				
Objective Aperture	none	100 µm	none		
Magnification	130,000 (EFTEM)	75,000	130,000 (EFTEM)		
Calibrated Pixel Size	0.946 Å/Px	1.064 Å/Px	0.946 Å/Px		
Beam diameter	1.3 µm	1 µm	1.2 µm	1.2 µm	1.2 µm
Total Exposure	70 $e^-/Å^2$	73 $e^-/Å^2$	70 $e^-/Å^2$	70 $e^-/Å^2$	70 $e^-/Å^2$
Exposure time	6.1 s	5.2 s	5.6s	5.6s	6.6s
Exposures per hole	1	1	1	3	3
Exposures per stage position	Unknown Ca. 60-150	5	Unknown ca. 60-150		
Acquisition at stage position	Fast (AFIS)	Image shift without beam tilt compensation	Fast (AFIS)		
Target range of under focus	0.5-1.4 µm	1.4-2.6 µm	0.5-1.2 µm	0.7-1.4 µm	0.5-1.2 µm
Movies in processing	41,392	19,325	22,191	19,865	28,915
Motion correction	CryoSPARC 4.4 Life patch-motion	Motion Cor2	CryoSPARC 4.6 Life patch-motion		
Picked particles	7,650,753	1,206,880	3,953,356	8,149,209	5,108,934
Maps and models in Supplementary Figure 2 were derived from the image acquisitions as indicated					
Conformation I, pH 4.0	Supplementary Table 2				
Conformation II, pH 4.0	Supplementary Table 3				
Conformation II, pH 8.0, phosphate			Supplementary Table 4		
Conformation II, pH 8.0, HEPES					Supplementary Table 5
Conformation III, pH 8.0			Supplementary Table 6		
Conformation I, pH 6.5		Supplementary Table 7			
Conformation II, pH 6.5		Supplementary Table 7			

Supplementary Table 2 | Cryo-EM data collection, refinement and validation statistics-Conformation I, pH4

	LifA conf.1 Composite (EMDB-19987) (PDB 9EUV)	LifA conf.1 Consensus (EMDB-19982)	LifA conf.1 Focused on C-term (EMDB-19983)
**Data collection and processing**			
Magnification		130,000	
Voltage (kV)		300	
Electron exposure (e–/Å^2)		70	
Defocus range (μm)		0.5-1.4	
Pixel size (Å)		0.946	
Symmetry imposed		C1	
Initial particle images (no.)		13,373,262	
Final particle images (no.)	440,787	449,787	120,944
Map resolution (Å)		2.7	3.0
FSC threshold: 0.143			
Map resolution range (Å)	2.5-3.5	2.7-4.5	2.7-3.9
**Refinement**			
Initial model used (PDB code)	-		
Model resolution (Å)	2.9		
FSC threshold	0.5		
Model resolution range (Å)			
Map sharpening *B* factor (Å^2)			
Model composition			
Non-hydrogen atoms	22287		
Protein residues	2796		
Ligands	-		
B factors (Å^2)			
Protein	105		
Ligand	-		
R.m.s. deviations			
Bond lengths (Å)	0.002		
Bond angles (°)	0.438		
Validation			
MolProbity score	1.57		
Clashscore	5.99		
Poor rotamers (%)	1.73		
Ramachandran plot			
Favored (%)	97.7		
Allowed (%)	2.3		
Disallowed (%)	0.0		

Supplementary Table 3 | Cryo-EM data collection, refinement and validation statistics, conformation II, pH4

	LifA conf.2 Composite (EMDB-19988) (PDB 9EUW)	LifA conf.2 Consensus (EMDB-19984)	LifA conf.2 Focused on C-term (EMDB-19985)
**Data collection and processing**			
Magnification	130,000		
Voltage (kV)	300		
Electron exposure (e–/Å^2)	70		
Defocus range (μm)	0.5-1.4		
Pixel size (Å)	0.946		
Symmetry imposed	-		
Initial particle images (no.)	13,373,262		
Final particle images (no.)		627,040	182,154
Map resolution (Å)		2.8	3.8
FSC threshold: 0.143			
Map resolution range (Å)	2.5-3.5	2.5-4.7	3.3-4.3
**Refinement**			
Initial model used (PDB code)	9EUV		
Model resolution (Å)	2.8		
FSC threshold	0.5		
Model resolution range (Å)			
Map sharpening *B* factor (Å^2)			
Model composition			
Non-hydrogen atoms	22324		
Protein residues	2801		
Ligands	-		
B factors (Å^2)			
Protein	106		
Ligand	-		
R.m.s. deviations			
Bond lengths (Å)	0.003		
Bond angles (°)	0.545		
Validation			
MolProbity score	1.9		
Clashscore	8.2		
Poor rotamers (%)	2.6		
Ramachandran plot			
Favored (%)	97.1		
Allowed (%)	2.8		
Disallowed (%)	0.0		

Supplementary Table 4 | Cryo-EM data collection, refinement and validation statistics-Conformation II, pH8, phosphate-buffer

	LifA conf.2 pH8, phosphate Composite Map (EMDB-53169) (PDB 9QHH)	LifA conf.2 pH8, phosphate (EMD-53168) Consensus	LifA conf.2 pH8, phosphate (EMD-53167) focused N-term	LifA conf.2 pH8, phosphate (EMD-53166) focused centre	LifA conf.2 pH8, phosphate (EMD-53165) focused S1	LifA conf.2 pH8, phosphate (EMD-53164) focused C-term
**Data collection and processing**						
Magnification				130,000		
Voltage (kV)				300		
Electron exposure (e–/Å^2)				70		
Defocus range (μm)				0.5-1.2		
Pixel size (Å)				0.946		
Symmetry imposed				C1		
Initial particle images (no.)				13,254,143		
Final particle images (no.)		1,294,835	921,427	490721	682,120	75,190
Map resolution (Å); FSC threshold: 0.143		3.2	3.1	3.1	3.1	4.4
Map resolution range (Å)	3.1-4.4	3.0-3.8	2.9-3.4	2.9-3.6	2.9-3.8	4.2-4.6
**Refinement**						
Initial model used (PDB code)	9EUW					
Model resolution (Å)	3.2					
FSC threshold	0.5					
Model resolution range (Å)						
Map sharpening *B* factor (Å^2)						
Model composition						
Non-hydrogen atoms	22339					
Protein residues	2803					
Ligands	-					
B factors (Å^2)						
Protein	80					
Ligand	-					
R.m.s. deviations						
Bond lengths (Å)	0.006					
Bond angles (°)	1.11					
Validation						
MolProbity score	1.96					
Clashscore	16					
Poor rotamers (%)	0.8					
Ramachandran plot						
Favored (%)	96.2					
Allowed (%)	3.65					
Disallowed (%)	0.18					

Supplementary Table 5 | Cryo-EM data collection, refinement and validation statistics-Conformation II, pH8, HEPES-buffer

	LifA conf.2 pH8, HEPES Composite Map (EMDB-52990) (PDB 9QB8)	LifA conf.2 pH8, HEPES (EMD-52985) Consensus	LifA conf.2 pH8, HEPES (EMD-52986 focused N-term	LifA conf.2 pH8, HEPES (EMD-52987) focused centre	LifA conf.2 pH8, HEPES (EMD-52988) focused S1	LifA conf.2 pH8, HEPES (EMD-52989) focused C-term
**Data collection and processing**						
Magnification				130,000		
Voltage (kV)				300		
Electron exposure (e–/Å^2)				70		
Defocus range (μm)				0.5-1.1		
Pixel size (Å)				0.946		
Symmetry imposed				C1		
Initial particle images (no.)				5,108,934		
Final particle images (no.)		298,552	181,550	139,319	185,230	70,995
Map resolution (Å); FSC threshold: 0.143		3.3	3.3	3.3	3.3	4.2
Map resolution range (Å)	3.3-4.2	3.1-4.7	3.2-3.8	3.1-4.1	3.2-4.0	4.0-4.5
**Refinement**						
Initial model used (PDB code)	9EUW					
Model resolution (Å)	3.3					
FSC threshold	0.5					
Model resolution range (Å)						
Map sharpening *B* factor (Å^2)						
Model composition						
Non-hydrogen atoms	22339					
Protein residues	2803					
Ligands	-					
B factors (Å^2)						
Protein	132					
Ligand	130					
R.m.s. deviations						
Bond lengths (Å)	0.004					
Bond angles (°)	0.66					
Validation						
MolProbity score	1.72					
Clashscore	10.3					
Poor rotamers (%)	0.5					
Ramachandran plot						
Favored (%)	96.9					
Allowed (%)	3.15					
Disallowed (%)	0					

Supplementary Table 6 | Cryo-EM data collection, refinement and validation statistics-Conformation III, pH8

	LifA conf.3 pH8 Composite Map (EMDB-52996) (PDB 9QBB)	LifA conf.3 pH8 (EMD-52995) Consensus	LifA conf.3 pH8 (EMD-52994 focused N-term	LifA conf.3 pH8 (EMD-52992) focused centre	LifA conf.3 pH8 (EMD-52991) focused S1	LifA conf.3 pH8 (EMD-52993) focused C-term
**Data collection and processing**						
Magnification				130,000		
Voltage (kV)				300		
Electron exposure (e–/Å^2)				70		
Defocus range (μm)				0.5-1.5		
Pixel size (Å)				0.946		
Symmetry imposed				C1		
Initial particle images (no.)				17,207,499		
Final particle images (no.)		219,576	164,449	80,295	77,207	139,166
Map resolution (Å); FSC threshold: 0.143		3.3	3.4	4.2	4.1	3.6
Map resolution range (Å)	3.3-4.2	3.0-5.4	3.2-4.9	4.0-7.4	3.9-5.4	3.4-4.5
**Refinement**						
Initial model used (PDB code)	9EUW					
Model resolution (Å)	3.3					
FSC threshold	0.5					
Model resolution range (Å)						
Map sharpening *B* factor (Å^2)						
Model composition						
Non-hydrogen atoms	23257					
Protein residues	2911					
Ligands	-					
B factors (Å^2)						
Protein	145					
Ligand						
R.m.s. deviations						
Bond lengths (Å)	0.005					
Bond angles (°)	1.07					
Validation						
MolProbity score	1.87					
Clashscore	10.9					
Poor rotamers (%)	0.19					
Ramachandran plot						
Favored (%)	95.5					
Allowed (%)	4.5					
Disallowed (%)	0					

Supplementary Table 7 | Cryo-EM data collection, refinement and validation statistics-Conformation I and II , pH 6.5

	LifA conf.1<br>pH 6.5<br>Consensus<br>(EMDB-53286)	LifA conf.2<br>pH 6.5<br>Consensus<br>(EMDB-53287)
**Data collection and processing**		
Magnification	75000	
Voltage (kV)	300	
Electron exposure (e–/Å^2)	73	
Defocus range (μm)	1.4-2.6	
Pixel size (Å)	1.064	
Symmetry imposed	C1	
Initial particle images (no.)	1,129,188	
Final particle images (no.)	168,300	216,178
Map resolution (Å)	3.5	3.4
FSC threshold: 0.143		
Map resolution range (Å)	2.9-7.1	2.9-6.0
**Refinement**		
Initial model used (PDB code)		
Model resolution (Å)		
FSC threshold		
Model resolution range (Å)		
Map sharpening *B* factor (Å^2)		
Model composition		
Non-hydrogen atoms		
Protein residues		
Ligands		
B factors (Å^2)		
Protein		
Ligand	No models built	
R.m.s. deviations		
Bond lengths (Å)		
Bond angles (°)		
Validation		
MolProbity score		
Clashscore		
Poor rotamers (%)		
Ramachandran plot		
Favored (%)		
Allowed (%)		
Disallowed (%)		

Supplementary References

1. Forsberg BO, Shah PNM, Burt A. A robust normalized local filter to estimate compositional heterogeneity directly from cryo-EM maps. *Nat Commun* **14**, 5802 (2023).
2. van Kempen M*, et al.* Fast and accurate protein structure search with Foldseek. *Nature Biotechnology* **42**, 243-246 (2023).
3. Meng EC*, et al.* UCSF ChimeraX: Tools for structure building and analysis. *Protein Sci* **32**, e4792 (2023).
4. Chen B, Liu Z, Perry K, Jin R. Structure of the glucosyltransferase domain of TcdA in complex with RhoA provides insights into substrate recognition. *Sci Rep* **12**, 9028 (2022).
5. Liu J*, et al.* Novel structural insights for a pair of monoclonal antibodies recognizing non-overlapping epitopes of the glucosyltransferase domain of Clostridium difficile toxin B. *Curr Res Struct Biol* **4**, 96-105 (2022).
6. Ziegler MO, Jank T, Aktories K, Schulz GE. Conformational changes and reaction of clostridial glycosylating toxins. *J Mol Biol* **377**, 1346-1356 (2008).
7. Malito E*, et al.* Structural basis for lack of toxicity of the diphtheria toxin mutant CRM197. *Proc Natl Acad Sci U S A* **109**, 5229-5234 (2012).
8. Shen A*, et al.* Defining an allosteric circuit in the cysteine protease domain of Clostridium difficile toxins. *Nat Struct Mol Biol* **18**, 364-371 (2011).
9. Belyy A, Heilen P, Hagel P, Hofnagel O, Raunser S. Structure and activation mechanism of the Makes caterpillars floppy 1 toxin. *Nat Commun* **14**, 8226 (2023).
10. Ravulapalli R*, et al.* Characterization of Vis Toxin, a Novel ADP-Ribosyltransferase from Vibrio splendidus. *Biochemistry* **54**, 5920-5936 (2015).
11. Punjani A, Zhang H, Fleet DJ. Non-uniform refinement: adaptive regularization improves single-particle cryo-EM reconstruction. *Nat Methods* **17**, 1214-1221 (2020).
12. Asarnow D, Palovcak E, Cheng Y. UCSF pyem v0.5. Zenodo. (2019).
13. Kimanius D*, et al.* Data-driven regularization lowers the size barrier of cryo-EM structure determination. *Nat Methods* **21**, 1216-1221 (2024).
14. Liebschner D*, et al.* Macromolecular structure determination using X-rays, neutrons and electrons: recent developments in Phenix. *Acta Crystallogr D Struct Biol* **75**, 861-877 (2019).
