# Supplementary figures and images for: Structure of lymphostatin, a large multi-functional virulence factor of pathogenic Escherichia coli
Source: Nat Commun. 2025 Jun 25;16:5389. doi: 10.1038/s41467-025-60995-9 (PMC12198386; doi:10.1038/s41467-025-60995-9)

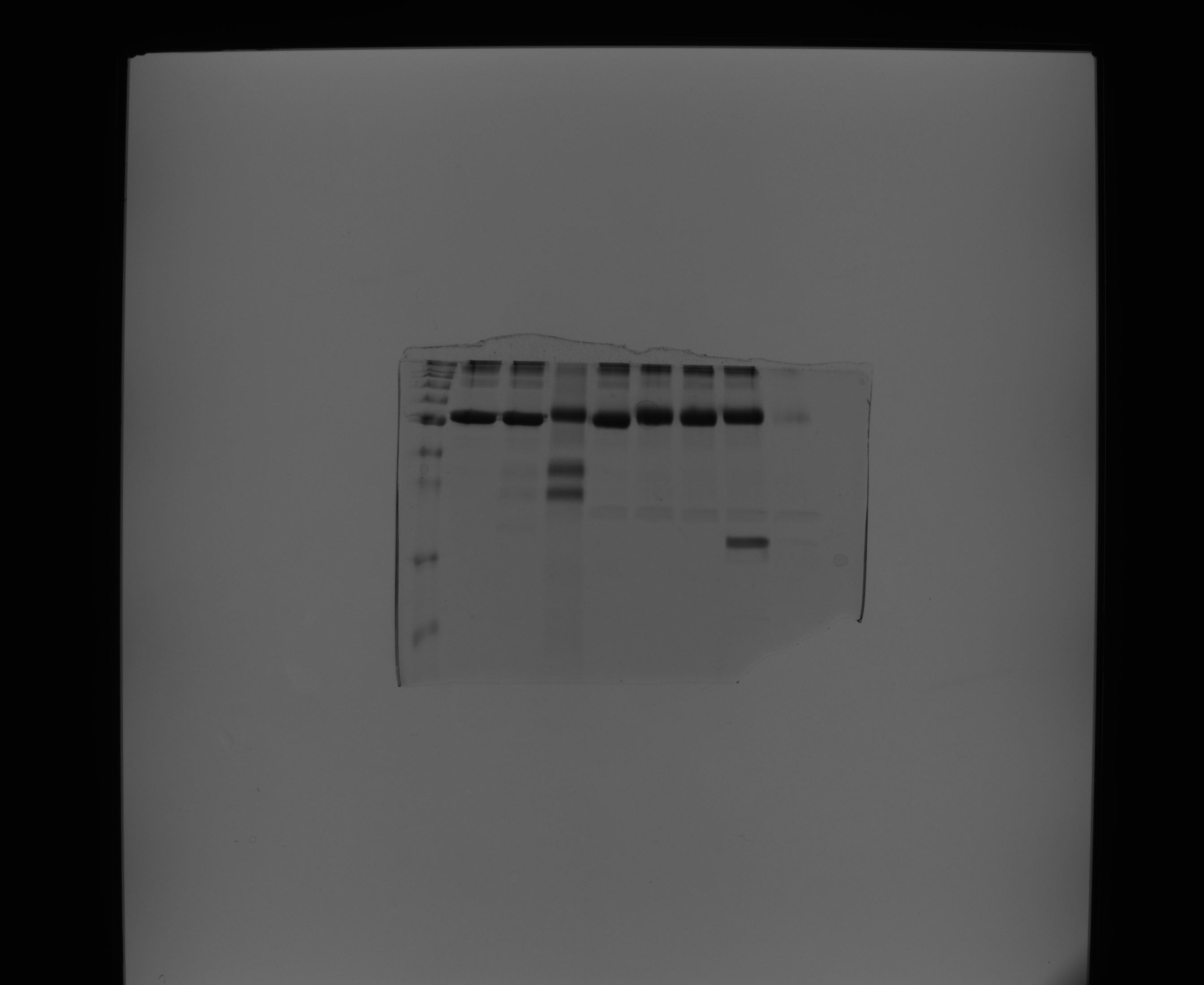

Supplement: Supplementary file 4 — Source Data [file 41467_2025_60995_MOESM4_ESM.zip › Figure3e.Tif]

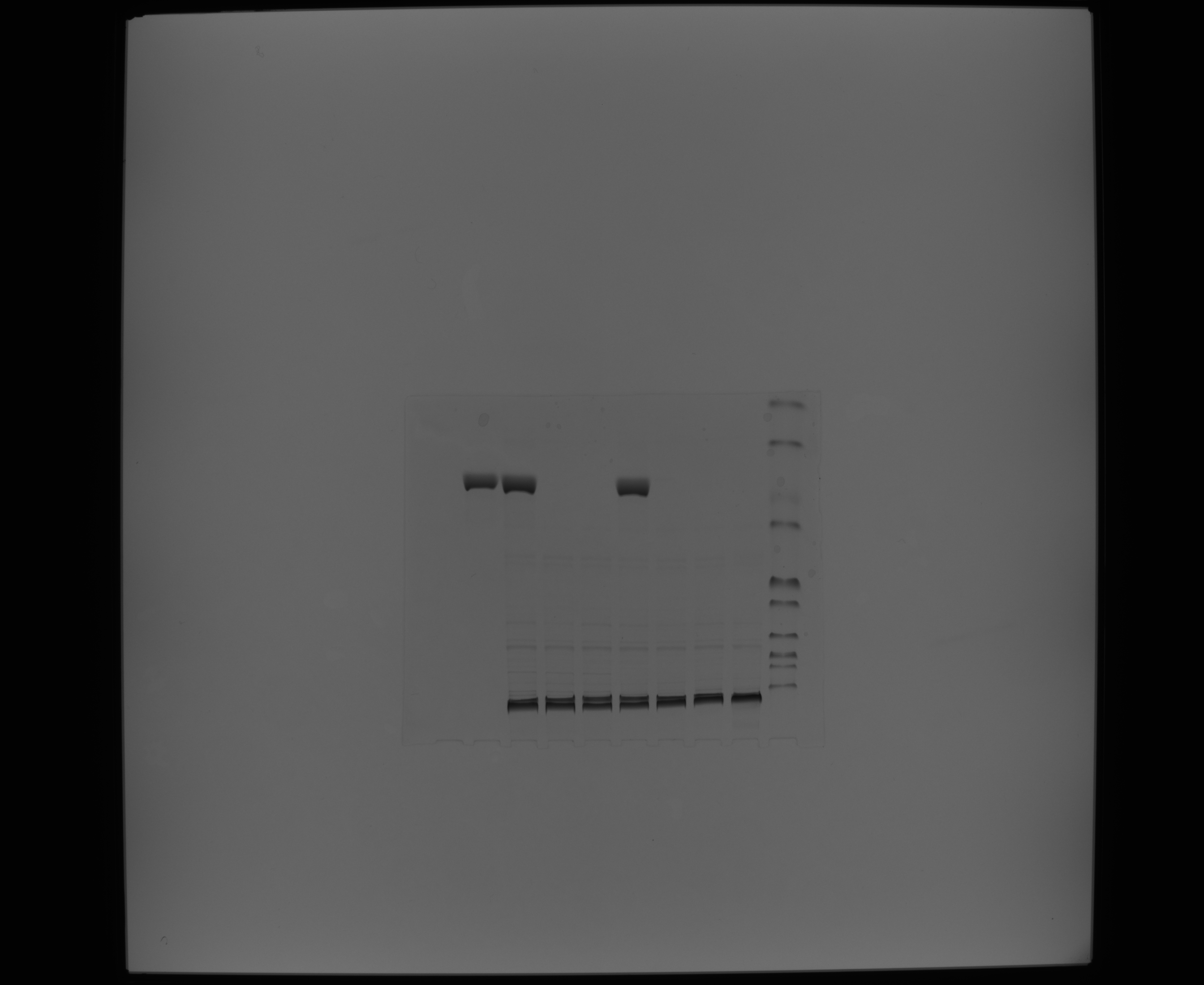

Supplement: Supplementary file 4 — Source Data [file 41467_2025_60995_MOESM4_ESM.zip › Figure3f_and_S10a_pH5-5.Tif]

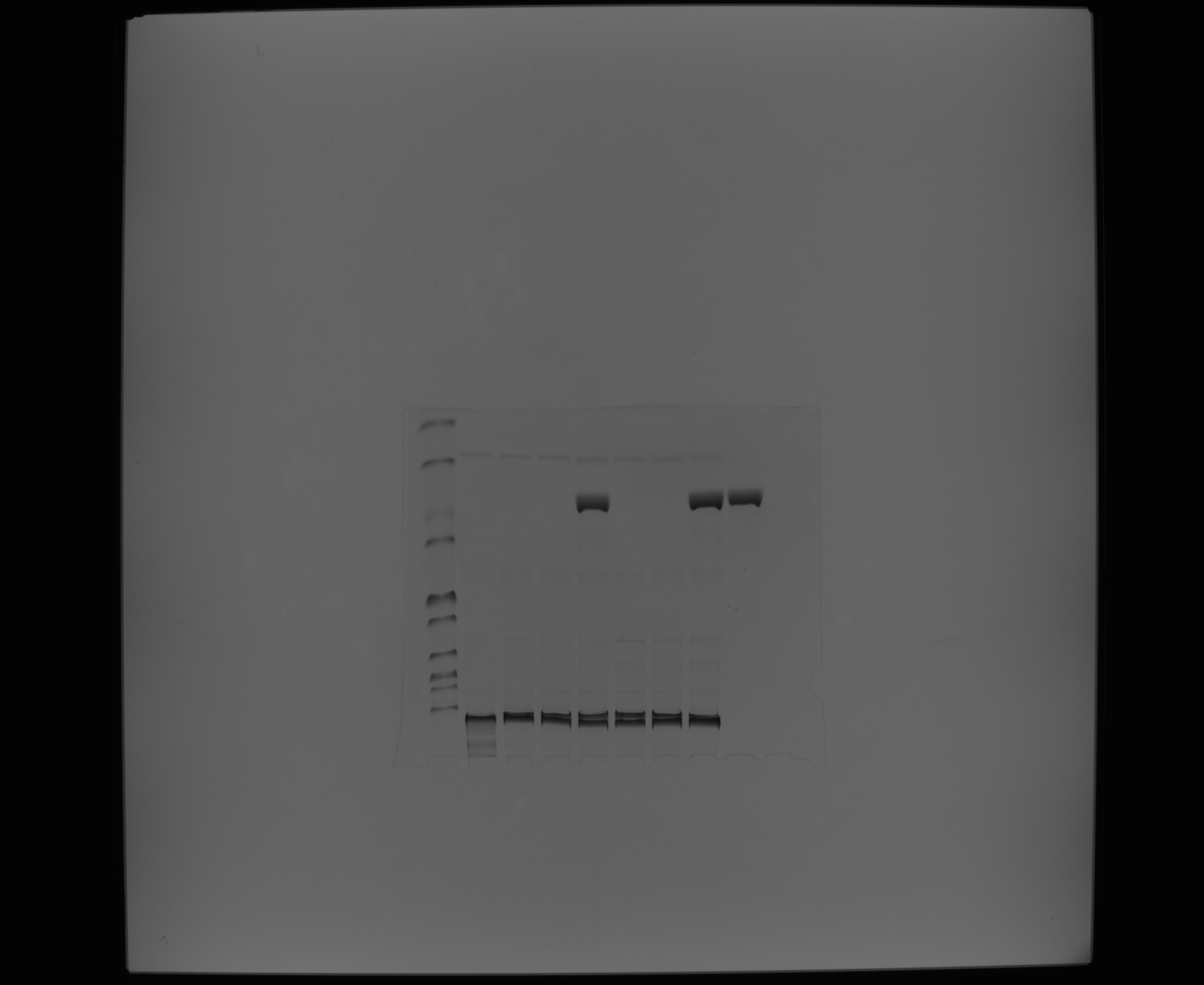

Supplement: Supplementary file 4 — Source Data [file 41467_2025_60995_MOESM4_ESM.zip › Figure3f_and_S10a_pH8-0.Tif]

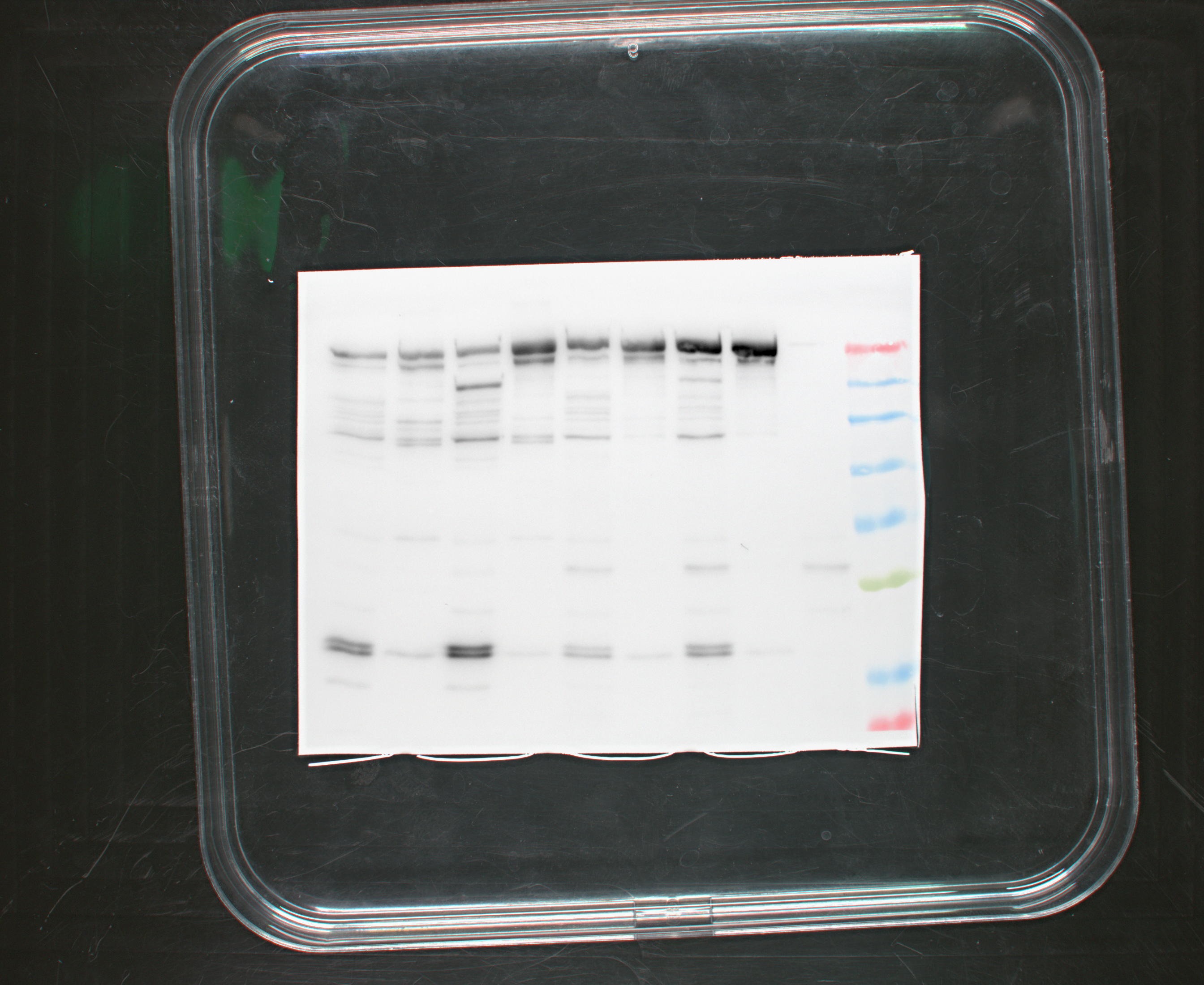

Supplement: Supplementary file 4 — Source Data [file 41467_2025_60995_MOESM4_ESM.zip › Figure3g_and_S10b.Tif]

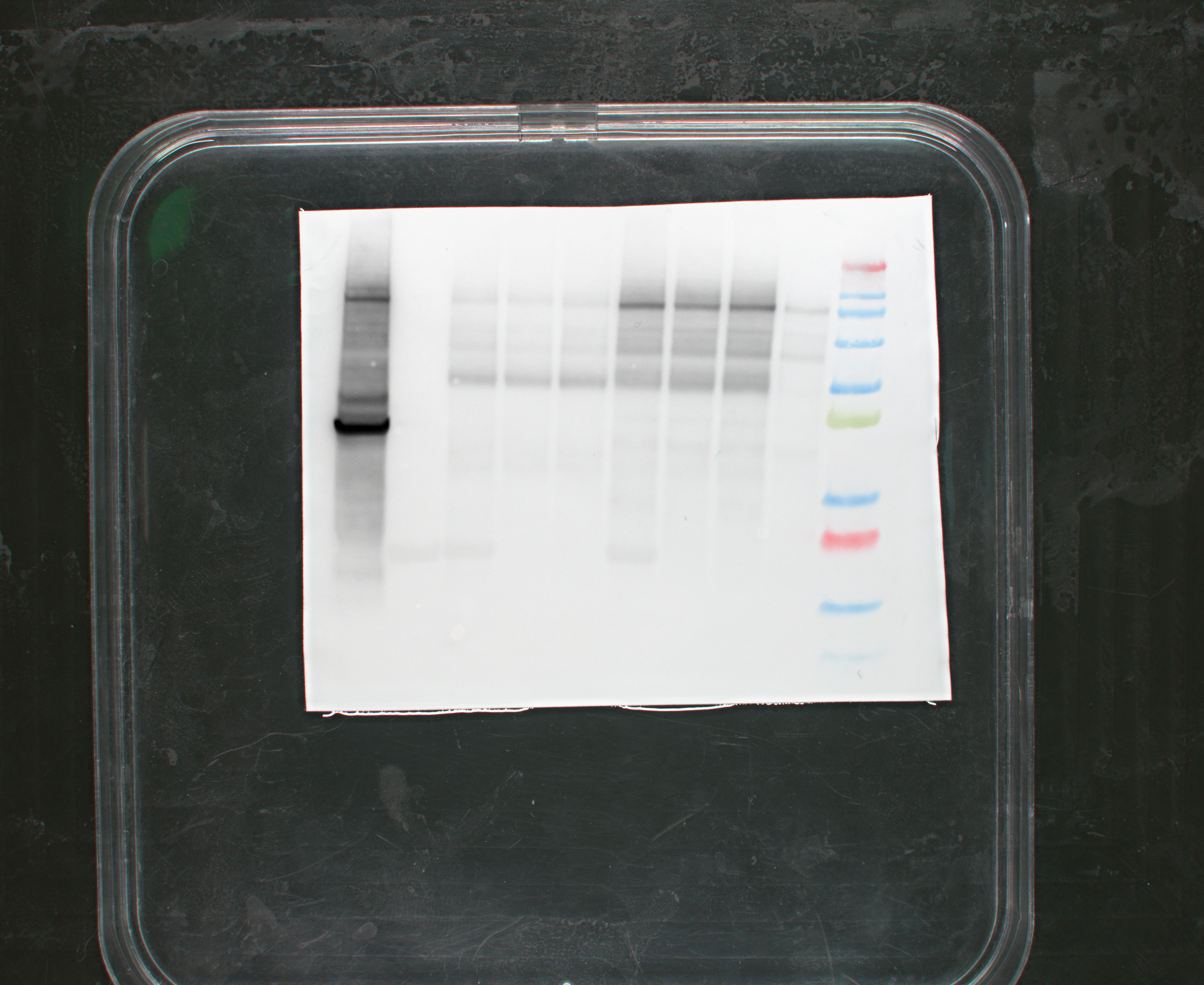

Supplement: Supplementary file 4 — Source Data [file 41467_2025_60995_MOESM4_ESM.zip › Figure5b_and_S14d.Tif]

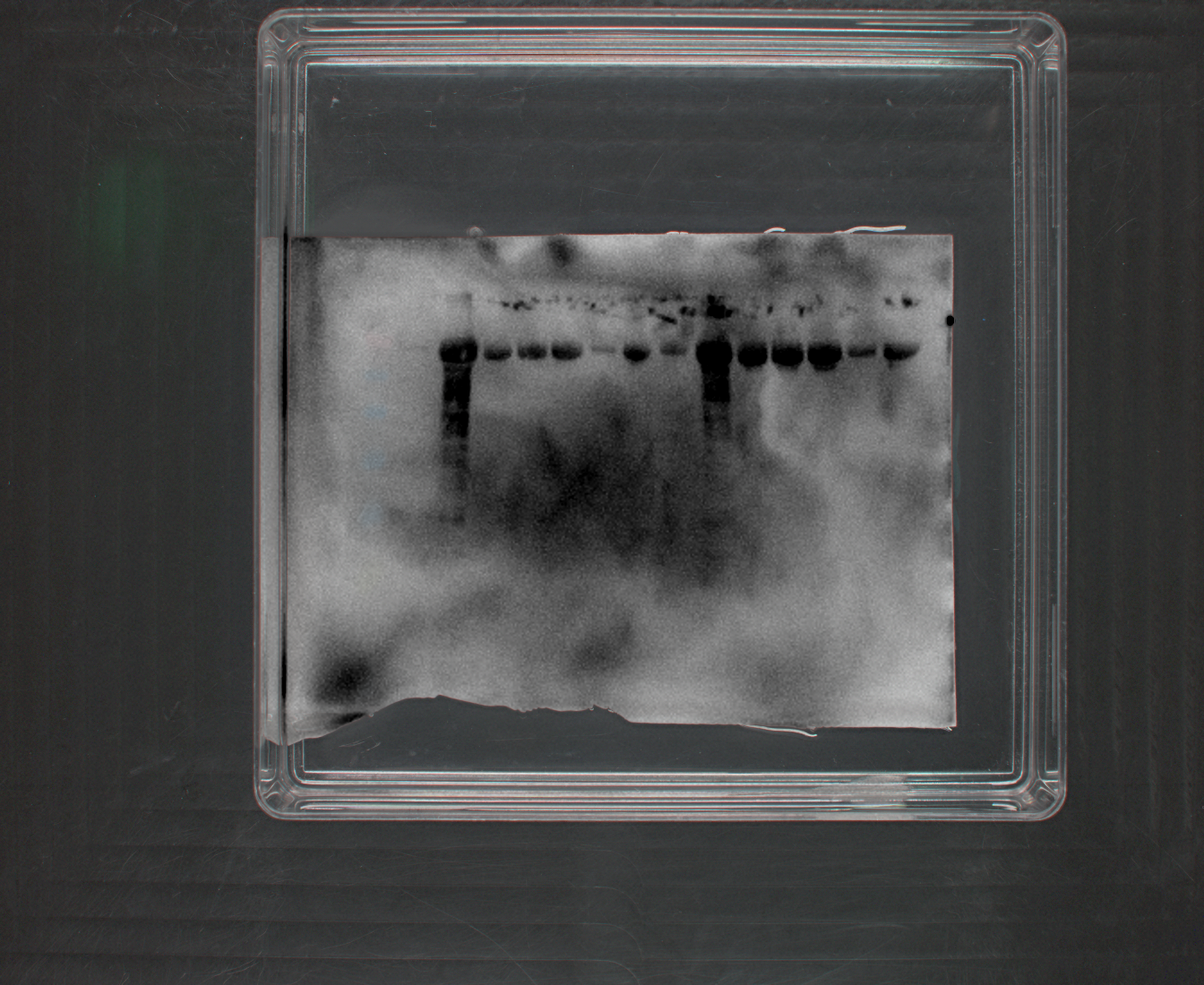

Supplement: Supplementary file 4 — Source Data [file 41467_2025_60995_MOESM4_ESM.zip › FigureS2a_blot.tif]

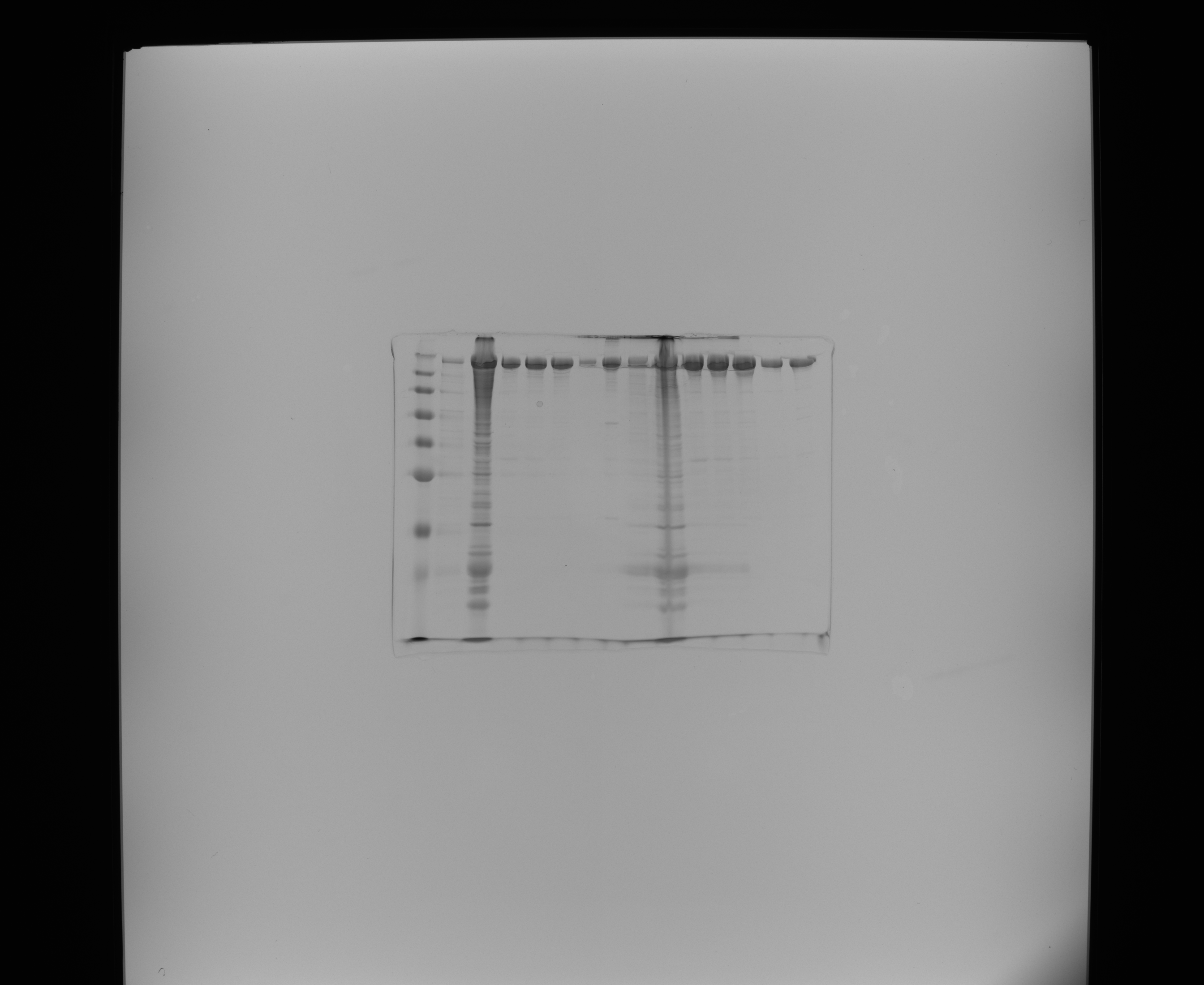

Supplement: Supplementary file 4 — Source Data [file 41467_2025_60995_MOESM4_ESM.zip › FigureS2a_SDS.tif]

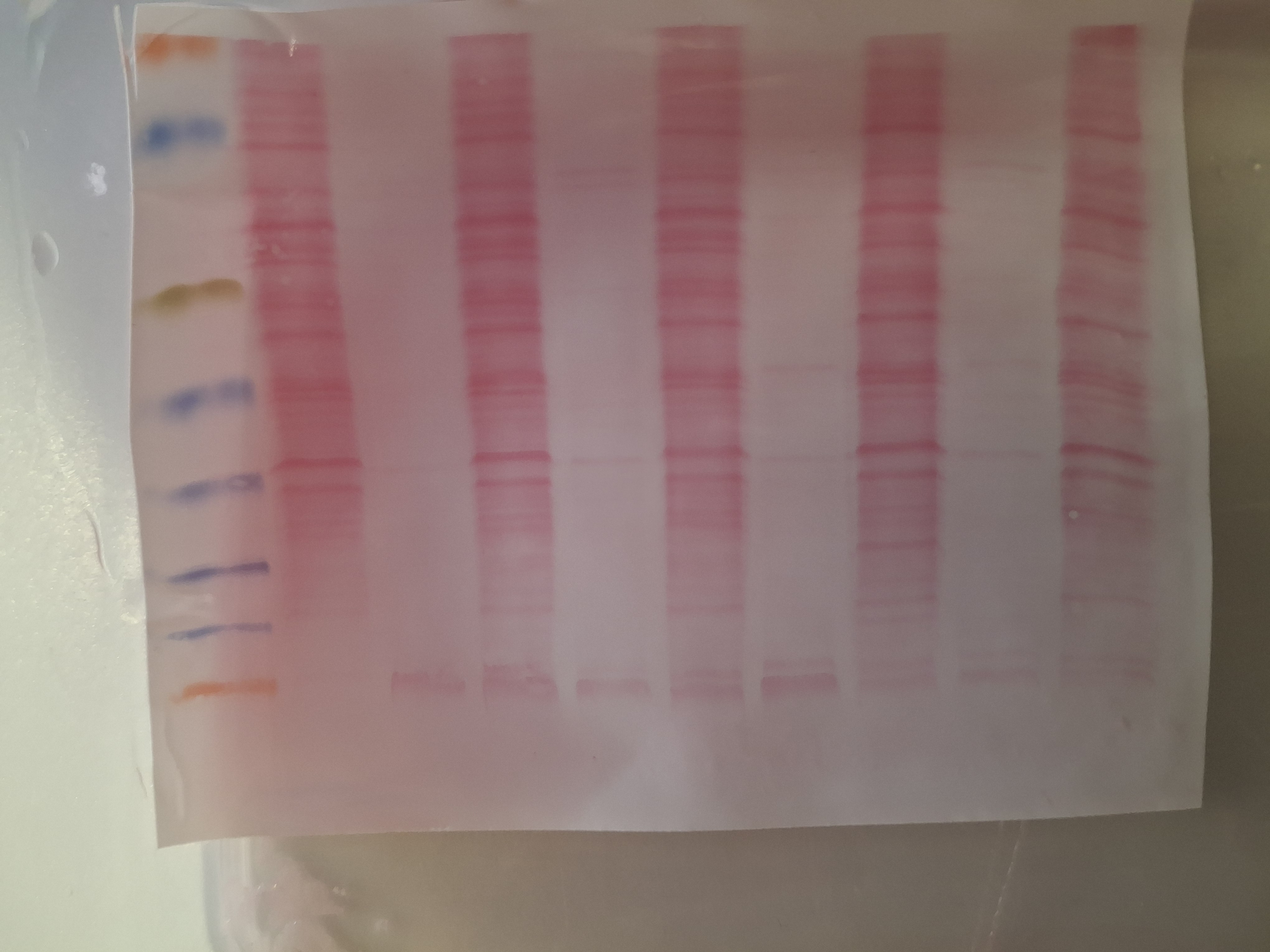

Supplement: Supplementary file 4 — Source Data [file 41467_2025_60995_MOESM4_ESM.zip › FigureS10b_PonceauS.jpg]

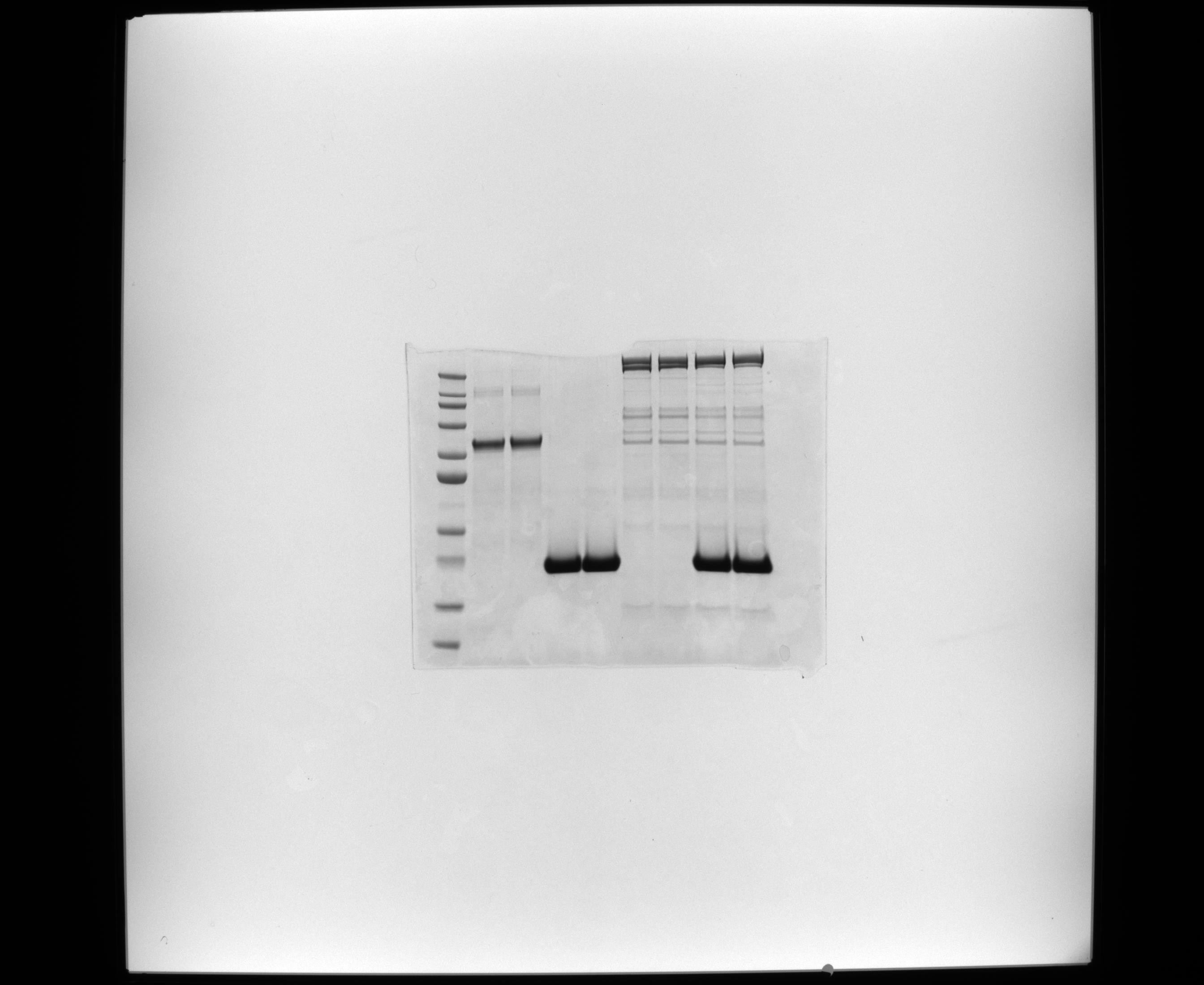

Supplement: Supplementary file 4 — Source Data [file 41467_2025_60995_MOESM4_ESM.zip › FigureS14b.Tif]

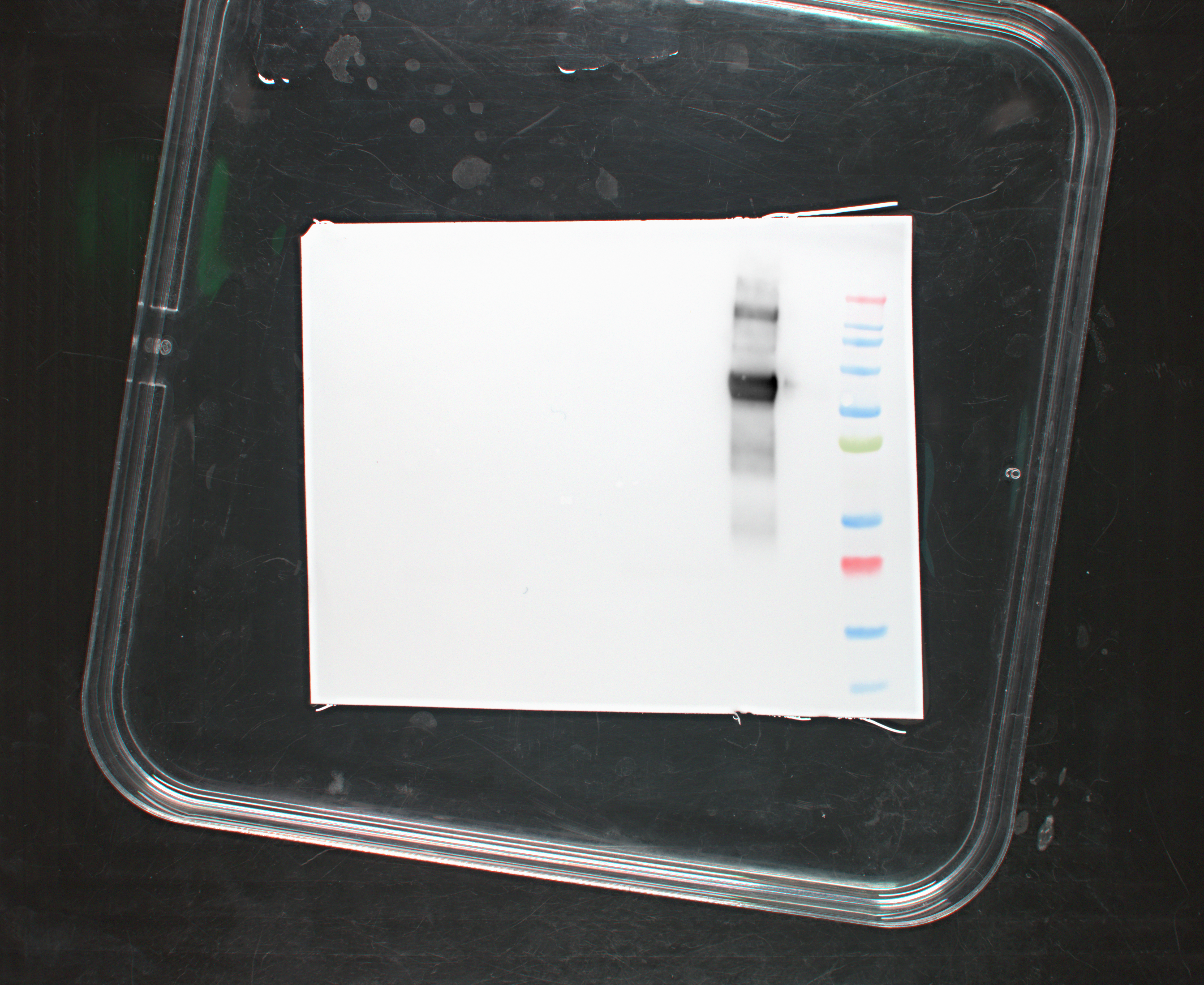

Supplement: Supplementary file 4 — Source Data [file 41467_2025_60995_MOESM4_ESM.zip › FigureS14c.Tif]

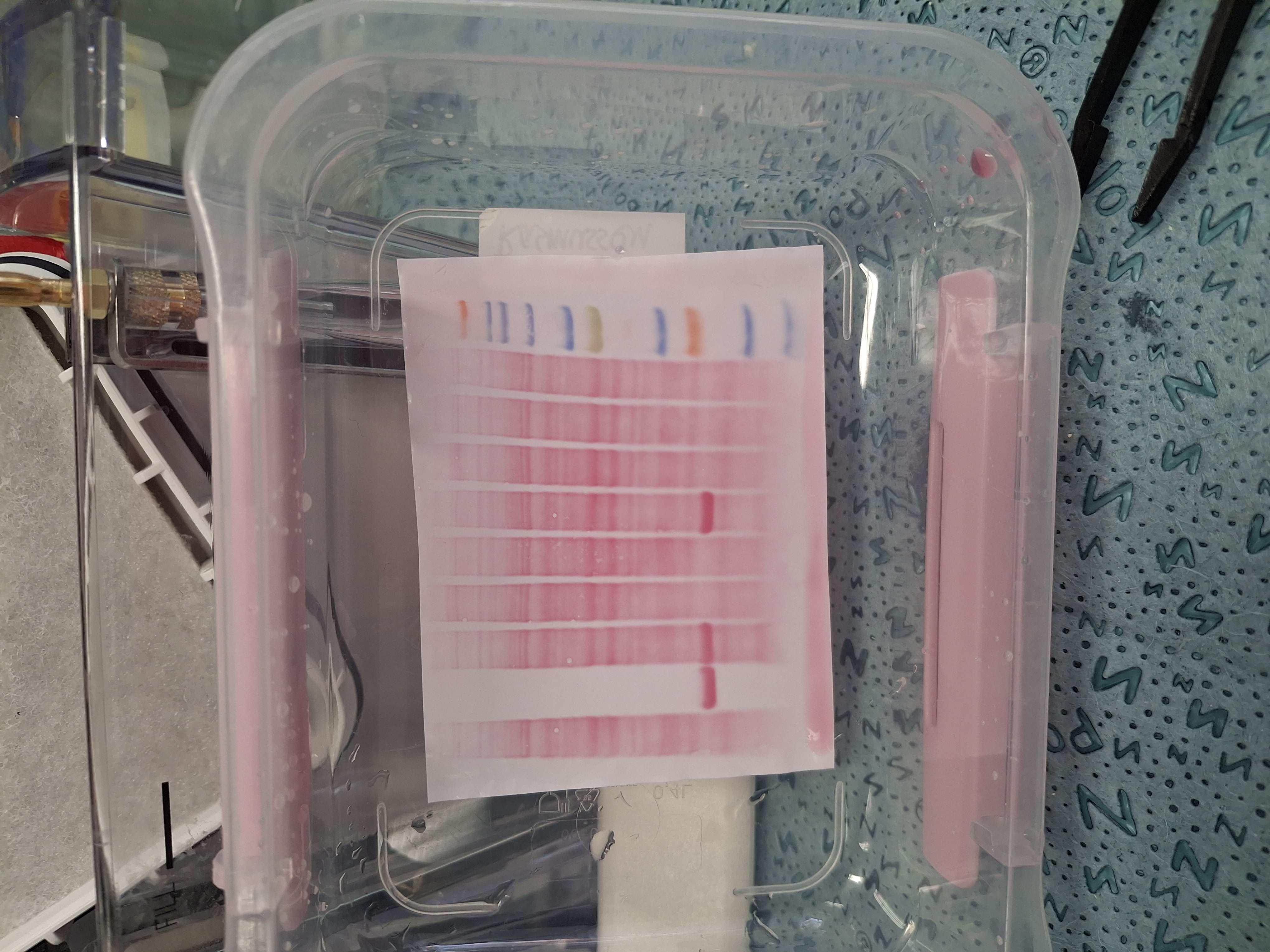

Supplement: Supplementary file 4 — Source Data [file 41467_2025_60995_MOESM4_ESM.zip › FigureS14e_PonceauS.jpg]
